# Low-Molecular-Weight Peptides from *Musca domestica* Larvae Alleviate Diarrhea-Predominant Irritable Bowel Syndrome Comorbid with Depression via Regulating Gut Microbiota, Short-Chain Fatty Acids and Serum Metabolism

**DOI:** 10.3390/ijms27167419

**Published:** 2026-08-19

**Authors:** Xiao Zhang, Xiaobao Jin, Hongyan Ma, Fujiang Chu

**Affiliations:** 1 School of Basic Medical Sciences, Guangdong Pharmaceutical University, Guangzhou Higher Education Mega Center, Guangzhou 510006, China; 18833081727@163.com; 2 Animal Experiment Center, Guangdong Pharmaceutical University, Guangzhou Higher Education Mega Center, Guangzhou 510006, China; jinxf2001@163.com; 3 School of Traditional Chinese Medicine, Guangdong Pharmaceutical University, Guangzhou Higher Education Mega Center, Guangzhou 510006, China; mahongyan@gdpu.edu.cn

**Keywords:** IBS-D, depression, gut microbiota, low-molecular-weight peptides, short-chain fatty acids, serum metabolomics, microbiota–gut–brain axis

## Abstract

Diarrhea-predominant irritable bowel syndrome (IBS-D) frequently occurs alongside depression, greatly impairing patients’ life quality, with limited targeted treatments. Low-molecular-weight peptides (LMWPs) from *Musca domestica* larvae exert gut-protective bioactivities, but their efficacy and molecular mechanisms for comorbid IBS-D and depression remain unelucidated. A chronic–acute combined stress (CACS)-induced rat model of IBS-D complicated with depression was established and treated with LMWPs. The study evaluated intestinal and depressive behavioral phenotypes, and integrated 16S rRNA sequencing, untargeted serum metabolomics and quantitative detection of fecal SCFAs for multi-omics correlation analysis. 1. CACS triggered typical comorbid symptoms, which LMWPs alleviated with effects similar to trimebutine maleate. 2. LMWP treatment was associated with reshaped gut microbiota, restored metabolic pathways, altered levels of 27 disease-associated serum metabolites, and increased fecal SCFA levels. 3. Multi-omics correlation analyses suggested that the therapeutic benefits may involve crosstalk among gut microbiota, SCFAs, and tryptophan metabolites. Larva-derived LMWP relieves IBS-D combined with depression by modulating gut microecology, SCFA generation and serum metabolic balance through the gut–brain axis, which can serve as a novel promising therapeutic candidate.

## 1. Introduction

Housefly (*Musca domestica*) larvae have been an important resource in traditional Chinese medicine and have been used clinically to cure chronic malnutrition and indigestion in children, ecthyma and lip boils since the Ming Dynasty (1368 A.D.) in China. They are believed to have the ability to clear heat, reduce fever, treat Crohn’s disease, and strengthen the spleen and stomach [1]. *Musca domestica* larvae are thought to represent a high-quality protein source that could be used as a livestock feed [2]. Further research has focused on the protein peptides derived from *Musca domestica* larvae, which have demonstrated various beneficial effects such as anti-inflammatory [3], anti-diarrheal [4] antibacterial [5], antitumor [6], antioxidant [7], hypolipidemic and anti-atherosclerosis [8] properties. In response to the traditional anti-diarrheal function of *Musca domestica* larvae, we conducted relevant studies in the preliminary stage. Our previous research results showed that whole *Musca domestica* larvae powder could modulate the structure of gut microbiota and thus improve host health. Subsequently, high-molecular-weight peptide (HMWPs, >60 KD), middle-molecular-weight peptide (MMWPs, 30 KD–60 KD) and low-molecular-weight peptides (LMWPs, <30 KD) of *Musca domestica* larvae were separated by Sephadex G-75 in previous research. The anti-diarrhea results showed that LMWP was better than HMWP and MMWP. Further microbial diversity indices showed that the LMWP treatment group exhibited a higher number of bands [9]. Furthermore, mice with irritable bowel syndrome with diarrhea (IBS-D) were utilized to investigate the structural changes in the gut microbiota and the regulation of the 5-HT pathway. The results of 16S rRNA gene sequencing suggested that LMWPs could induce structural changes in the gut microbiota and alter the levels of specific gut microbiota, including Bacteroidetes, Proteobacteria, and Verrucomicrobia. LMWPs were found to potentially function by regulating 5-HT, SERT, 5-HT2AR, 5-HT3AR, and 5-HT4R, as indicated by the results of ELISA, qRT-PCR, and IHC [10].

It has been reported that up to 94% of IBS patients demonstrate psychiatric disorders, including somatoform disorders, major depression and anxiety [11]. Further studies have shown that the comorbidity rates of depression and anxiety are relatively high among patients with IBS-D, which may be related to the imbalance of the gut microbiota [12]. Microbes provide the host with a range of otherwise inaccessible metabolic capabilities [13]. Although amplicon sequencing characterizes the taxonomic composition of the gut microbiome, it is impossible to cover the direct evidence of the microbial biological functions related to the gut microbial community [14]. The integration of metabolomics and microbiomics offers a powerful approach to studying the intricate interactions between the microbiome and its host, revealing insights into health and disease [15]. By combining these two “omics” approaches, researchers can gain a more holistic understanding of the complex interplay between microbial activity and host physiology [16].

One key area where metabolomics and microbiomics integration is proving invaluable is in understanding the gut–brain axis [17].The gut microbiome can influence brain development and behavior through the production of metabolites that interact with the host’s nervous system [18]. Gut microbiota dysbiosis is highly significant in irritable bowel syndrome (IBS), particularly in diarrhea-predominant IBS (IBS-D), and is influenced by various triggers such as infections, diet, antibiotics, stress, and poor sleep [19]. These factors can alter the composition and diversity of the intestinal microbiota, leading to imbalances that contribute to IBS pathophysiology [20]. Studies have consistently shown that patients with IBS-D exhibit reduced gut microbial diversity and distinct microbial compositions compared to healthy individuals [21,22].

On the basis of our prior research and other relevant studies, we aim to further investigate the efficacy and underlying mechanisms by which low-molecular-weight peptides derived from *Musca domestica* larvae inhibit diarrhea-predominant irritable bowel syndrome (IBS-D) comorbid with depression through the integration of metabolomics and microbiomics approaches, with a focus on their regulatory effects on intestinal microecology.

## 2. Result

### 2.1. Identification of the Musca domestica Larvae Low-Molecule-Weight Peptides

**Figure 1:**
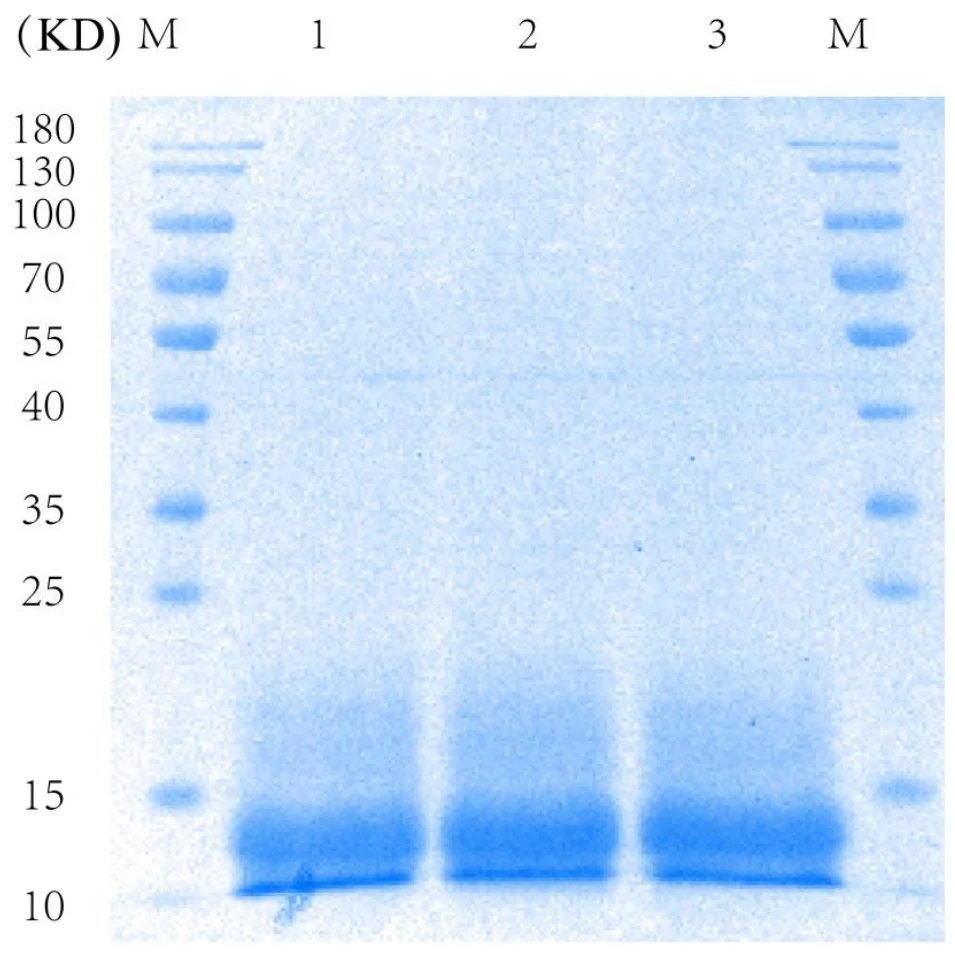
SDS-PAGE electrophoresis diagram of *Musca domestica* larvae low-molecular-weight peptides. Lanes 1, 2, and 3 represent the same batch of low-molecular-weight peptides from *Musca domestica* larvae, which were prepared via ultrafiltration.

The low-molecular-weight peptides (LMWPs) used in this experiment were prepared via the ultrafiltration centrifugation method, a patented technology of our research group. These peptides had a molecular weight of less than 30 kDa and a purity of more than 95%, which met the experimental requirements. The purity of LMWPs was verified by sodium dodecyl sulfate–polyacrylamide gel electrophoresis (SDS-PAGE) and analyzed using ImageJ software (version 1.8.0), as shown in Figure 1.

### 2.2. Effects of LMWPs on General Condition, Body Weight, Food Intake and Diarrhea in IBS-D Rats

**Figure 2:**
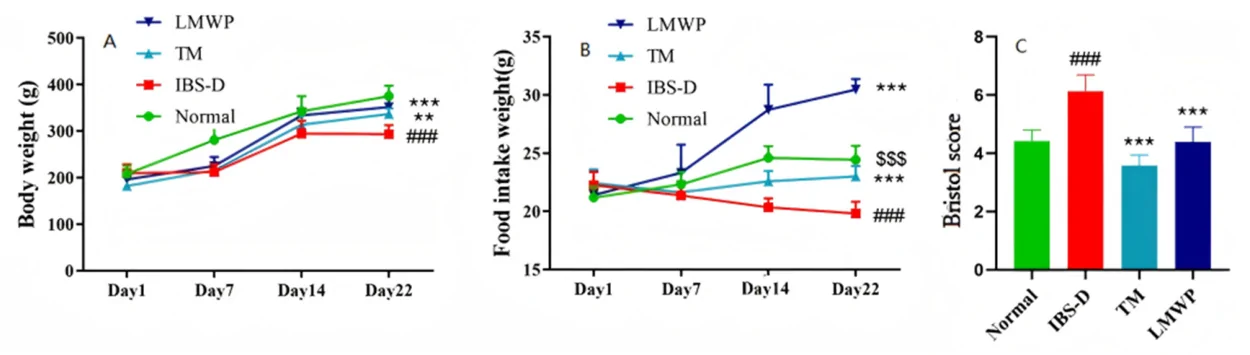
Changes in body weight, food intake and diarrhea in rats of each group. (**A**) Body weight changes of rats in each group. ^###^ *p* < 0.001 vs. Normal; ** *p* < 0.01, *** *p* < 0.001 vs. Model. (**B**) Changes in food intake of rats in each group. ^###^ *p* < 0.001 vs. Normal; *** *p* < 0.001 vs. Model; ^$$$^ *p* < 0.001 Normal vs. LMWP. (**C**) Changes in diarrhea of rats in each group. ^###^ *p* < 0.001 vs. Normal; *** *p* < 0.001 vs. Model. *n* = 8. Statistical significance was assessed using one-way ANOVA followed by Tukey’s post hoc test. Significant differences were observed among groups in final body weight, daily food intake, and Bristol stool score (*p* < 0.0001).

Rats in the IBS-D model group showed poor general physical condition, as qualitatively observed by reduced spontaneous activity, dull and unkempt fur, and heightened reactivity to handling. Quantitatively, these rats exhibited significantly decreased body weight (*p* < 0.001, Figure 2A), reduced food intake (*p* < 0.001, Figure 2B), and severe diarrhea (*p* < 0.001, Figure 2C). Additionally, these rats displayed decreased appetite, loose stools of varying severity, fur contaminated by feces, and a sparse, dull, and yellowish appearance. In contrast, rats in the TM (Trimebutin Maleate) group and LMWP (low-molecular-weight peptides from *Musca domestica* larvae) group showed significant recovery after pharmacological intervention, presenting with increased locomotor activity, improved mental status, normal dietary intake and defecation, and smooth and glossy fur. With respect to body weight changes, after 22 days of modeling, the body weight of rats in the IBS-D model group was significantly lower than that in the normal group (*p* < 0.001). Compared with the IBS-D model group, the body weights of rats in the TM treatment group and LMWP treatment group were increased to different extents, with statistically significant differences (*p* < 0.01 and *p* < 0.001, respectively). These results are illustrated in Figure 2A.

Regarding food intake, after 22 days of modeling, the food intake of the IBS-D model group was significantly lower than that of the normal group (*p* < 0.001). In comparison with the IBS-D model group, the food intake of rats in both the TM treatment group and LMWP treatment group was significantly elevated (*p* < 0.001 for both groups). Notably, the food intake of the LMWP treatment group was significantly higher than that of the normal group (*p* < 0.001). The corresponding results are presented in Figure 2B.

After chronic–acute combined stress (CACS) induction, the Bristol stool form score in the IBS-D model group was significantly higher than that in the normal group (*p* < 0.001). The Bristol stool form scores in the TM treatment group and LMWP treatment group were significantly lower than that in the IBS-D model group (*p* < 0.001), indicating that both the TM treatment and LMWP treatment could improve the diarrhea status of IBS-D rats, as shown in Figure 2C.

### 2.3. Effects of LMWPs on Visceral Sensitivity and Colonic Motility in IBS-D Rats

**Figure 3:**
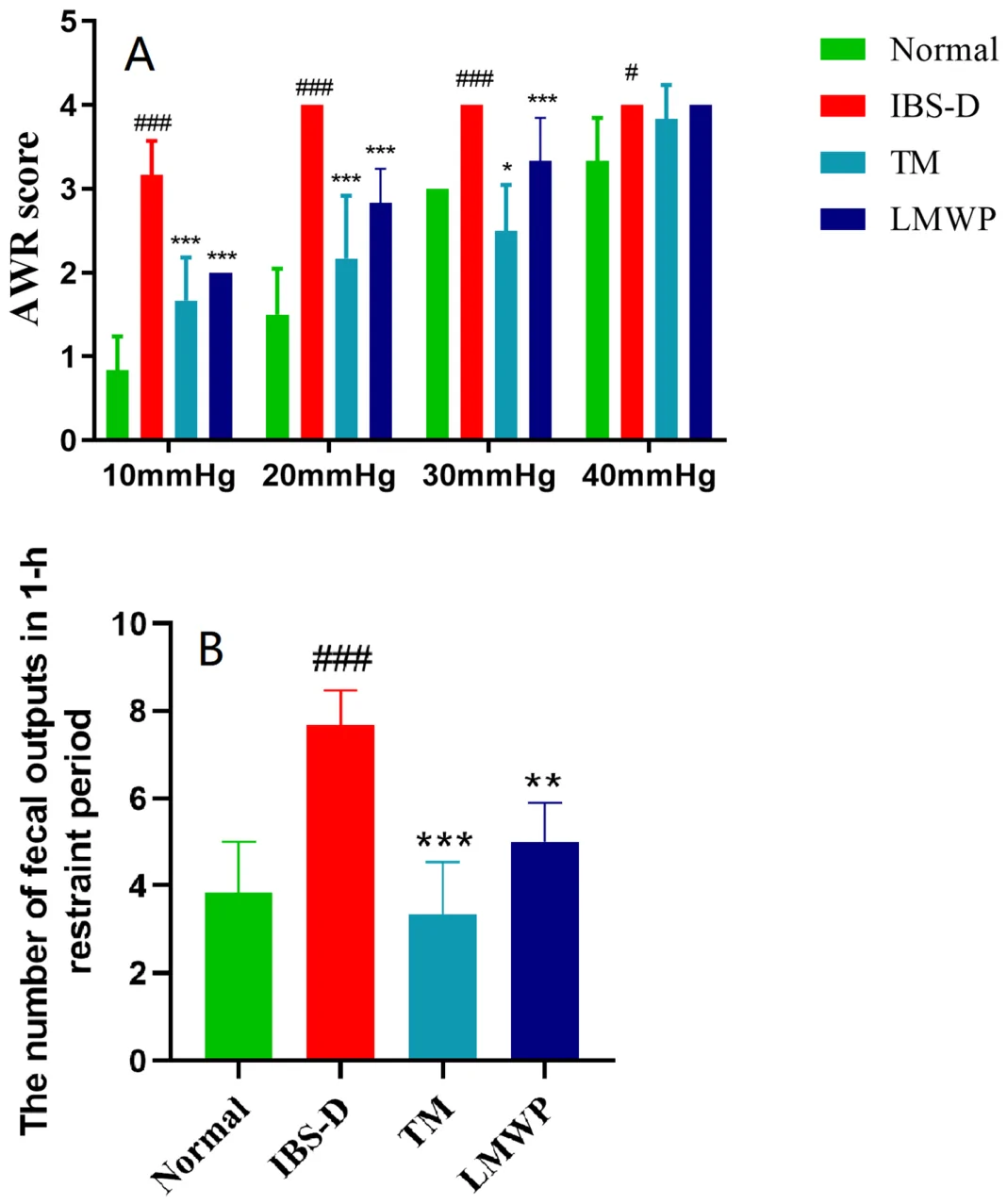
Effects of LMWP on visceral sensitivity and colonic motility in IBS-D rats. (**A**) Changes in visceral sensitivity of rats in each group. ^###^ *p* < 0.001 vs. Normal; ^#^ *p* < 0.05 vs. Normal; * *p* < 0.05 vs. Model; *** *p* < 0.001 vs. Model. (**B**) Colonic motility in rats in each group. ^###^ *p* < 0.001 vs. Normal; ** *p* < 0.01, *** *p* < 0.001 vs. Model. *n* = 8. Statistical significance was assessed using one-way ANOVA followed by Tukey’s post hoc test. Significant differences were observed among groups in AWR scores at pressures of 10 mmHg (*p* < 0.0001), 20 mmHg (*p* < 0.0001), 30 mmHg (*p* < 0.0001), and 40 mmHg (*p* < 0.05), as well as in 1 h colonic fecal output (*p* < 0.0001).

The results of visceral sensitivity showed that the AWR scores in the IBS-D model group were significantly higher than those in the normal group at distension pressures of 10 mmHg, 20 mmHg, 30 mmHg, and 40 mmHg (*p* < 0.001, *p* < 0.001, *p* < 0.001, *p* < 0.05, respectively). At 10 mmHg, the AWR scores in both the TM treatment group and LMWP treatment group were significantly decreased compared with the IBS-D model group (*p* < 0.001 and *p* < 0.001, respectively). At 20 mmHg, the AWR scores in both the TM treatment group and LMWP treatment group were also significantly lower than those in the IBS-D model group (*p* < 0.001 and *p* < 0.001, respectively). At 30 mmHg, the AWR scores in the TM treatment group and LMWP treatment group were significantly reduced compared with the IBS-D model group (*p* < 0.05 and *p* < 0.001, respectively). At 40 mmHg, the AWR score in the IBS-D model group remained significantly higher than that in the normal group (*p* < 0.05). Compared with the IBS-D model group, neither the TM treatment group nor the LMWP treatment group showed a statistically significant reduction in AWR score (both *p* > 0.05), as shown in Figure 3A.

The effect of LMWPs on colonic motor dysfunction was determined by measuring fecal output within 1 h on day 22. The fecal pellet output in the IBS-D model group was nearly two-fold higher than that in the normal group (*p* < 0.001). However, treatment with LMWPs significantly inhibited the increased fecal pellet output (*p* < 0.01), which was similar to the effect observed in the positive drug TM treatment group (*p* < 0.001), as shown in Figure 3B.

### 2.4. Effects of LMWPs on Depressive-like Behaviors in IBS-D Rats

**Figure 4:**
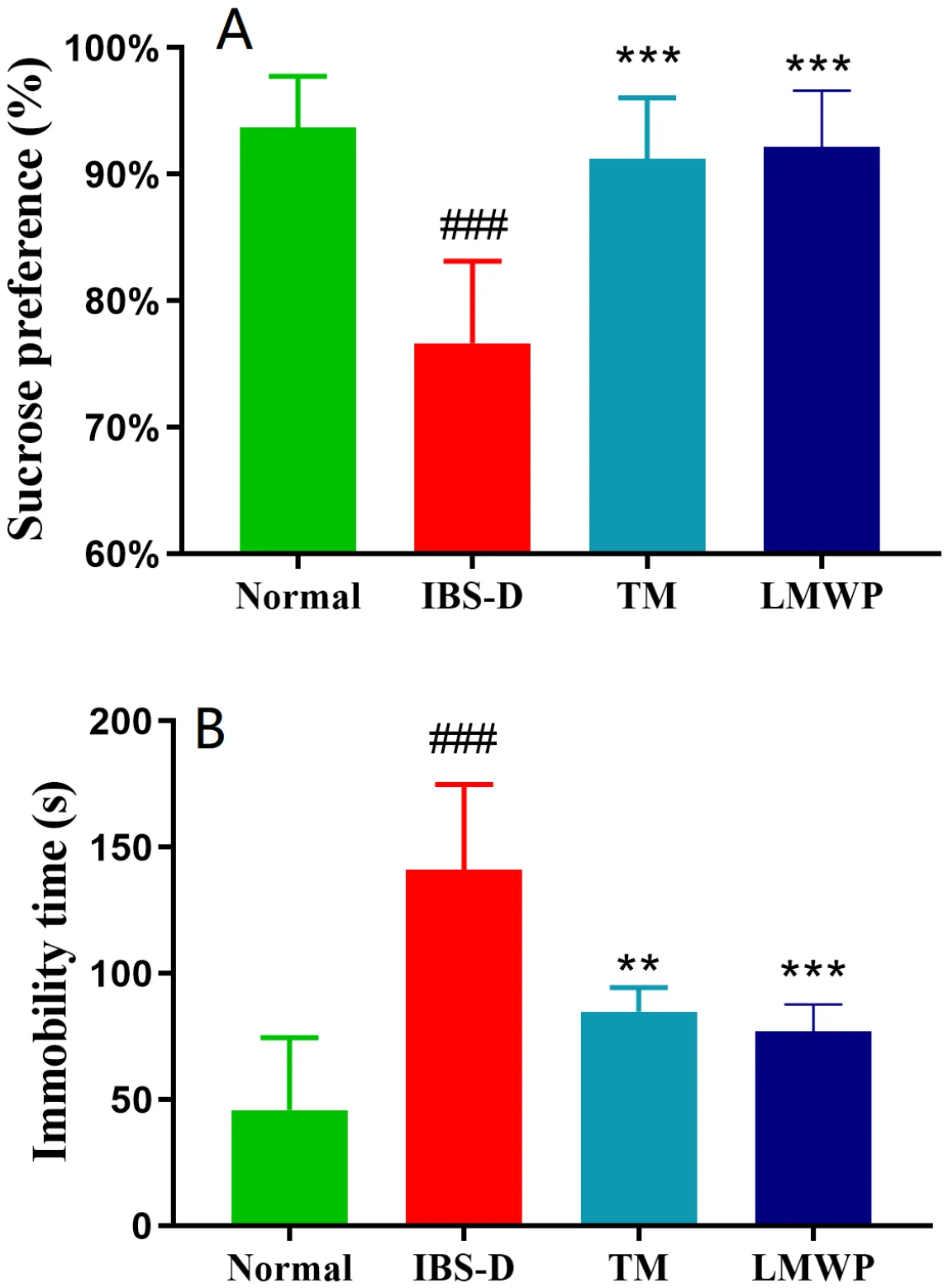
Effects of LMWP on depressive-like behaviors in IBS-D rats. (**A**) Sucrose preference of rats in each group. ^###^ *p* < 0.001 vs. Normal; *** *p* < 0.001 vs. Model. (**B**) Immobility time in the forced swimming test of rats in each group. ^###^ *p* < 0.001 vs. Normal; ** *p* < 0.01 vs. Model; *** *p* < 0.001 vs. Model. *n* = 8. Statistical significance was assessed using one-way ANOVA followed by Tukey’s post hoc test. Significant differences were observed among groups in sucrose preference and forced swimming tests (*p* < 0.0001).

The potential antidepressant-like effects of the LMWP treatment group and the TM treatment group were evaluated using the sucrose preference test and the forced swimming test. As shown in Figure 4A, the sucrose preference rate in the IBS-D model group was significantly decreased compared with the normal group (*p* < 0.001). Compared with the IBS-D model group, treatment with LMWP significantly increased the sucrose preference rate (*p* < 0.001), and the sucrose preference rate was also significantly elevated in the TM treatment group after treatment (*p* < 0.001).

In the forced swimming test, the immobility time of rats in the IBS-D model group was significantly longer than that in the normal group (*p* < 0.001). The immobility time of IBS-D rats treated with LMWPs was significantly shortened compared with the IBS-D model group (*p* < 0.001). Similarly, the TM treatment group also showed reduced immobility time compared with the IBS-D model group (*p* < 0.01), as shown in Figure 4B. These results indicate that LMWP treatment exerts antidepressant-like effects in the CACS-induced IBS-D rat model.

In the open-field test, relative to the normal group, the model group displayed marked decreases in both central zone travel distance and central zone residence time (*p* < 0.001). When compared with the model group, the LMWP and trimebutine maleate groups both showed increased central zone travel distance to varying extents (*p* < 0.05), and the LMWP group additionally exhibited significant prolongation of central zone residence time (*p* < 0.01) (Table 1).

### 2.5. Effects of LMWPs on Contents of 5-HT and BDNF in Hippocampus and Colonic Tissues of IBS-D Rats

Under light microscopy at 400× magnification, no significant differences were observed in the morphology and arrangement of neurons in the hippocampal dentate gyrus (DG) across all groups, with clear neuronal nucleoli visible. For colonic mucosa, the normal group showed no epithelial shedding, while the IBS-D model group exhibited disorganized or missing colonic tissue with mild mucosal epithelial damage (without severe pathological lesions). The TM and LMWP treatment groups showed relatively regular colonic tissue arrangement with rare epithelial loss.

**Figure 5:**
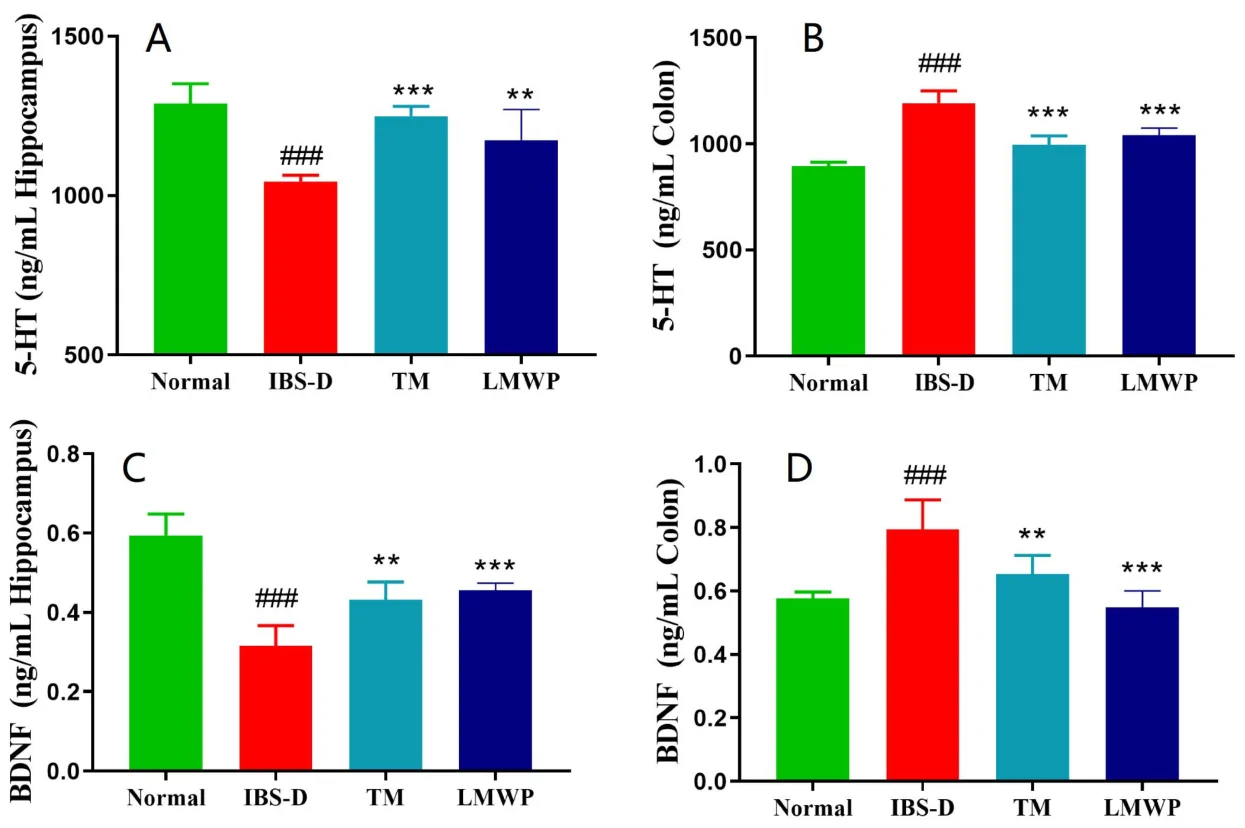
The effects of LMWPs on the contents of 5-HT and BDNF in hippocampus and colonic Tissues of IBS-D rats. (**A**,**B**) 5-HT content in hippocampal and colonic tissues of rats in each group. (**C**,**D**) BDNF content in hippocampal and colonic tissues of rats in each group. ^###^ *p* < 0.001 vs. Normal; ** *p* < 0.01 vs. Model; *** *p* < 0.001 vs. Model. *n* = 8. Statistical significance was assessed using one-way ANOVA followed by Tukey’s post hoc test. Significant differences were observed among groups in the levels of 5-HT and BDNF in the hippocampus and colon (*p* < 0.0001).

Hippocampal and colonic tissues were further collected, and the contents of 5-HT and BDNF were measured by ELISA. As shown in Figure 5, compared with the normal group, the IBS-D model group exhibited significantly increased 5-HT and BDNF levels in colonic tissues (both *p* < 0.001, Figure 5B,D), whereas both 5-HT and BDNF levels in the hippocampus were significantly decreased (both *p* < 0.001, Figure 5A,C). Compared with the IBS-D model group, the TM and LMWP treatments significantly reduced colonic 5-HT and BDNF concentrations (Figure 5B,D), while significantly increasing hippocampal 5-HT and BDNF levels (Figure 5A,C).

### 2.6. Effects of LMWPs on mRNA Expression Levels of 5-HT1AR, BDNF, PKA and CREB in Hippocampal and Colonic Tissues of IBS-D Rats

The results showed that, compared with the normal group, the relative mRNA expression levels of 5-HT1AR, BDNF, PKA, and CREB in the hippocampus were significantly decreased in the IBS-D model group (*p* < 0.01, *p* < 0.001, *p* < 0.05, and *p* < 0.05, respectively). Compared with the IBS-D model group, the LMWP treatment significantly increased hippocampal mRNA expression of 5-HT1AR, BDNF, PKA, and CREB (*p* < 0.001, *p* < 0.01, *p* < 0.001, and *p* < 0.001, respectively). Similarly, the TM treatment significantly increased hippocampal mRNA expression of 5-HT1AR, PKA, and CREB (*p* < 0.001, *p* < 0.01, and *p* < 0.001, respectively), while no significant change was observed in BDNF expression.

**Figure 6:**
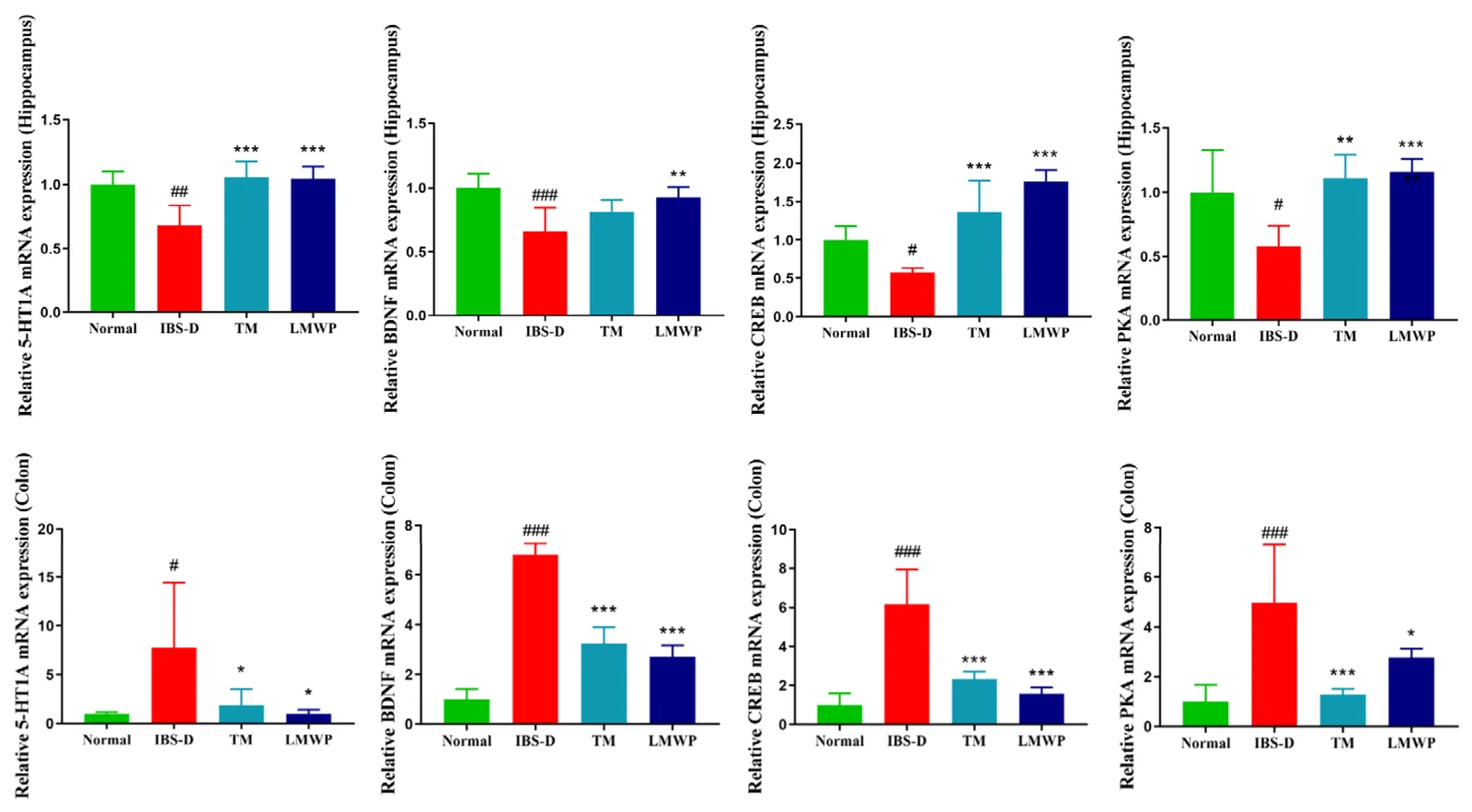
The effects of LMWPs on mRNA expression of 5-HT1AR, BDNF, PKA and CREB in hippocampal and colonic tissues of IBS-D rats. ^#^ *p* < 0.05, ^##^ *p* < 0.01, ^###^ *p* < 0.001 vs. Normal; * *p* < 0.05, ** *p* < 0.01, *** *p* < 0.001 vs. IBS-D model group. *n* = 6. Statistical significance was assessed using one-way ANOVA followed by Tukey’s post hoc test. Significant differences among groups were observed in the expression levels of all four tested genes in the hippocampus and colon (all *p* < 0.0001).

In colonic tissue, compared with the normal group, the relative mRNA expression levels of 5-HT1AR, BDNF, CREB, and PKA were significantly increased in the IBS-D model group (*p* < 0.05, *p* < 0.001, *p* < 0.001, and *p* < 0.001, respectively). Compared with the IBS-D model group, both LMWP and TM treatments significantly decreased colonic mRNA expression of all four genes. Specifically, in the LMWP group, the decreases were significant for 5-HT1AR (*p* < 0.05), BDNF (*p* < 0.001), CREB (*p* < 0.001), and PKA (*p* < 0.05). In the TM group, the decreases were significant for 5-HT1AR (*p* < 0.05), BDNF (*p* < 0.001), CREB (*p* < 0.001), and PKA (*p* < 0.001). The above results are shown in Figure 6.

### 2.7. Effects of LMWPs on Intestinal Microecological Diversity in IBS-D Rats

#### 2.7.1. Effects of LMWPs on Alpha and Beta Diversity of Intestinal Flora in IBS-D Rats

**Figure 7:**
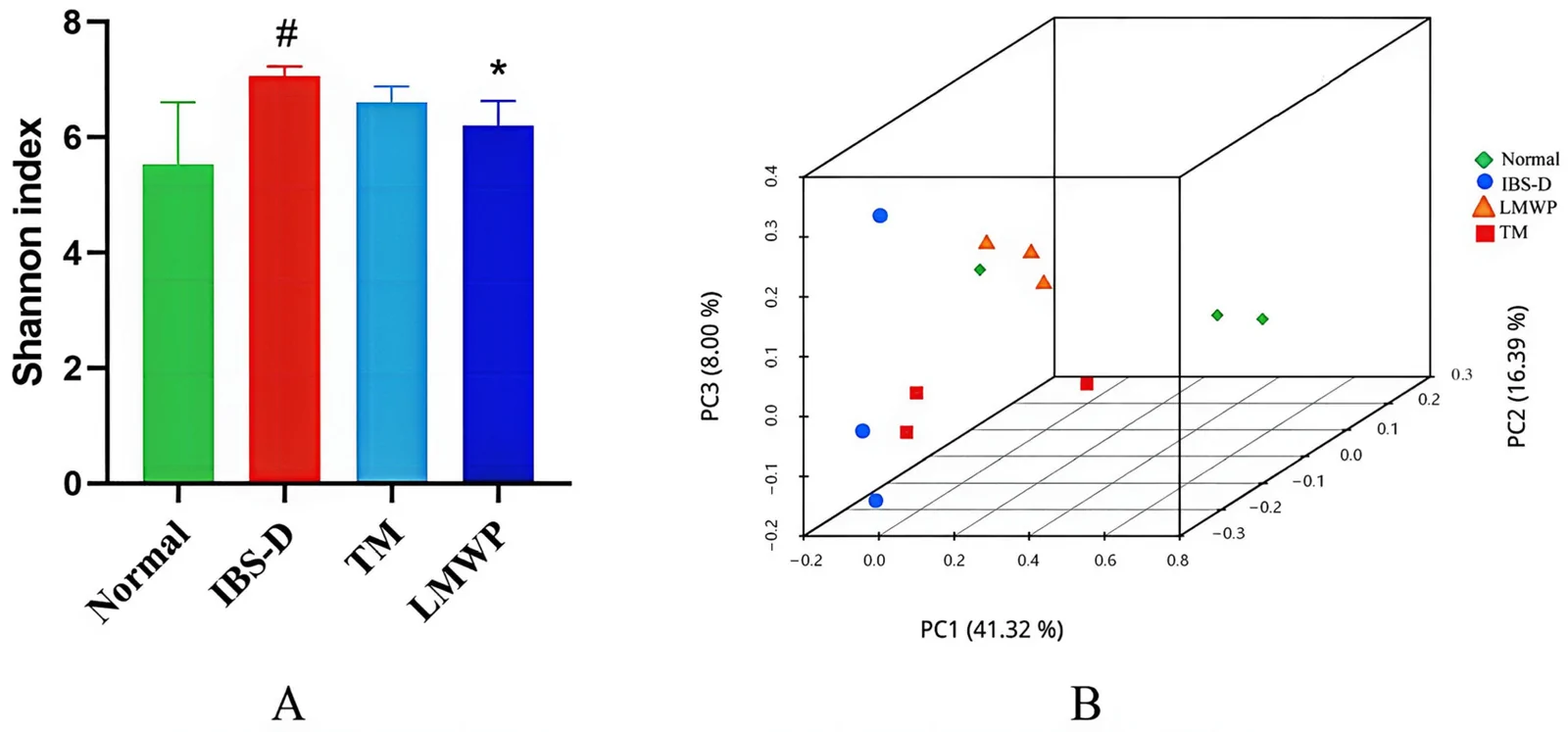
The effects of LMWPs on the alpha and beta diversity of intestinal flora in IBS-D rats. (**A**) Shannon index analysis of intestinal microbial alpha diversity in each group; (**B**) principal coordinates analysis (PCoA) of intestinal microbial beta diversity based on weighted UniFrac distance. ^#^ *p* < 0.05 vs. Normal; * *p* < 0.05 vs. IBS-D model group. *n* = 3.

As shown in Figure 7, compared with the normal control group, the Shannon index of the IBS-D model group was significantly increased (*p* < 0.05), and the two groups show distinct clustering separation in the PCoA plot with tight intragroup aggregation, indicating that IBS-D modeling significantly altered microbial alpha diversity and severely disrupted the overall community structure. Compared with the model group, the LMWP intervention significantly reduced the Shannon index (*p* < 0.05) but did not fully restore the sample distribution to the level of the normal control group in the PCoA plot. PERMANOVA demonstrated significant differences in overall microbial community structure among the four groups (F = 4.21, R^2^ = 0.42, *p* = 0.001), indicating that the LMWP intervention substantially altered the gut microbiota landscape.

#### 2.7.2. Effects of LMWPs on Intestinal Flora Diversity and Species Composition in IBS-D Rats

**Figure 8:**
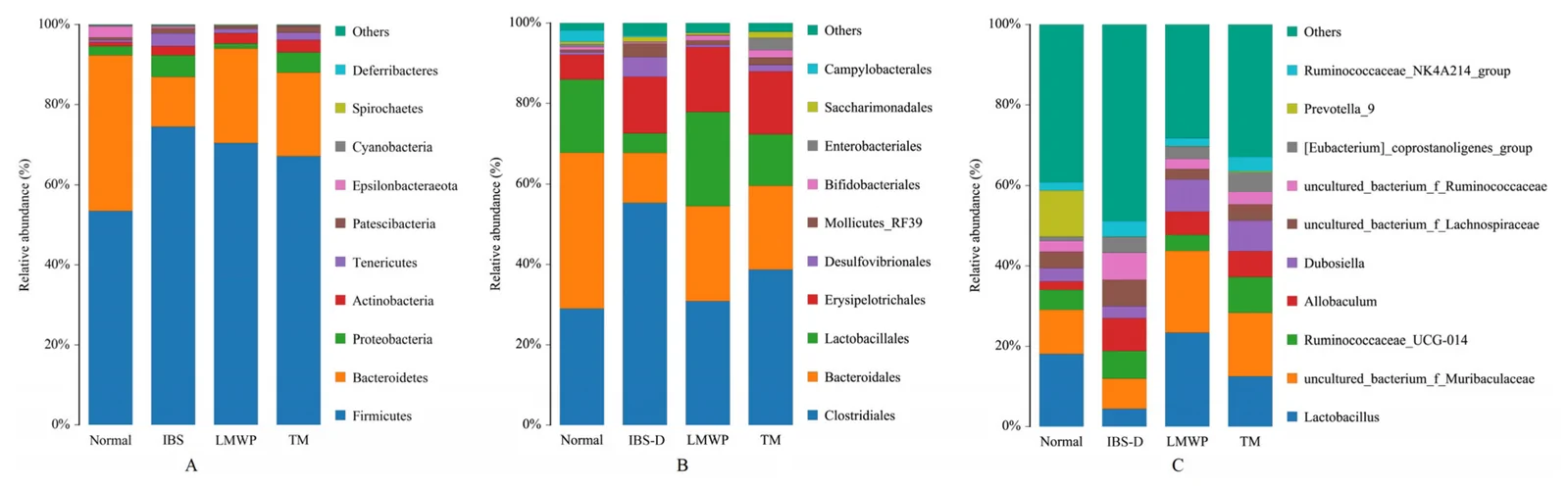
Effects of LMWP on intestinal flora diversity and species compositions in IBS-D rats. (**A**) Phylum-level taxonomic analysis of intestinal flora; (**B**) order-level taxonomic analysis of intestinal flora; (**C**) genus-level taxonomic analysis of intestinal flora.

The integrated analysis of sample sequences was performed to investigate the changes in the intestinal flora structure and relative abundance in IBS-D rats and LMWP-intervened IBS-D rats, as shown in Figure 8A–C. At the phylum level, compared with the normal control group, the relative abundance of Bacteroidetes was significantly decreased, while that of Firmicutes was increased in IBS-D rats, and LMWP treatment reversed these abnormal alterations; in addition, the Firmicutes/Bacteroidetes (F/B) ratio was notably higher in IBS-D rats than that in normal rats (q < 0.05), and both LMWP and trimebutine maleate interventions reduced the F/B ratio of IBS-D rats (q < 0.05). At the order level, the relative abundance of Clostridiales was elevated, whereas the abundances of Lactobacillales and Bacteroidales were decreased in IBS-D rats relative to the normal control group (q < 0.05), and these disordered changes at the order level were restored after treatment with LMWP and trimebutine maleate. At the genus level, compared with the normal control group, the relative abundances of Ruminococcaceae_NK4A214_group, uncultured_bacterium_f_Ruminococcaceae, uncultured_bacterium_f_Lachnospiraceae and Allobaculum were increased in IBS-D rats, while the abundances of Dubosiella, uncultured_bacterium_f_Muribaculaceae and Lactobacillus were decreased, and similarly, these abnormal changes at the genus level were reversed by LMWP and trimebutine maleate treatment.

#### 2.7.3. Effects of LMWPs on Functional Prediction of Intestinal Flora in IBS-D Rats

**Figure 9:**
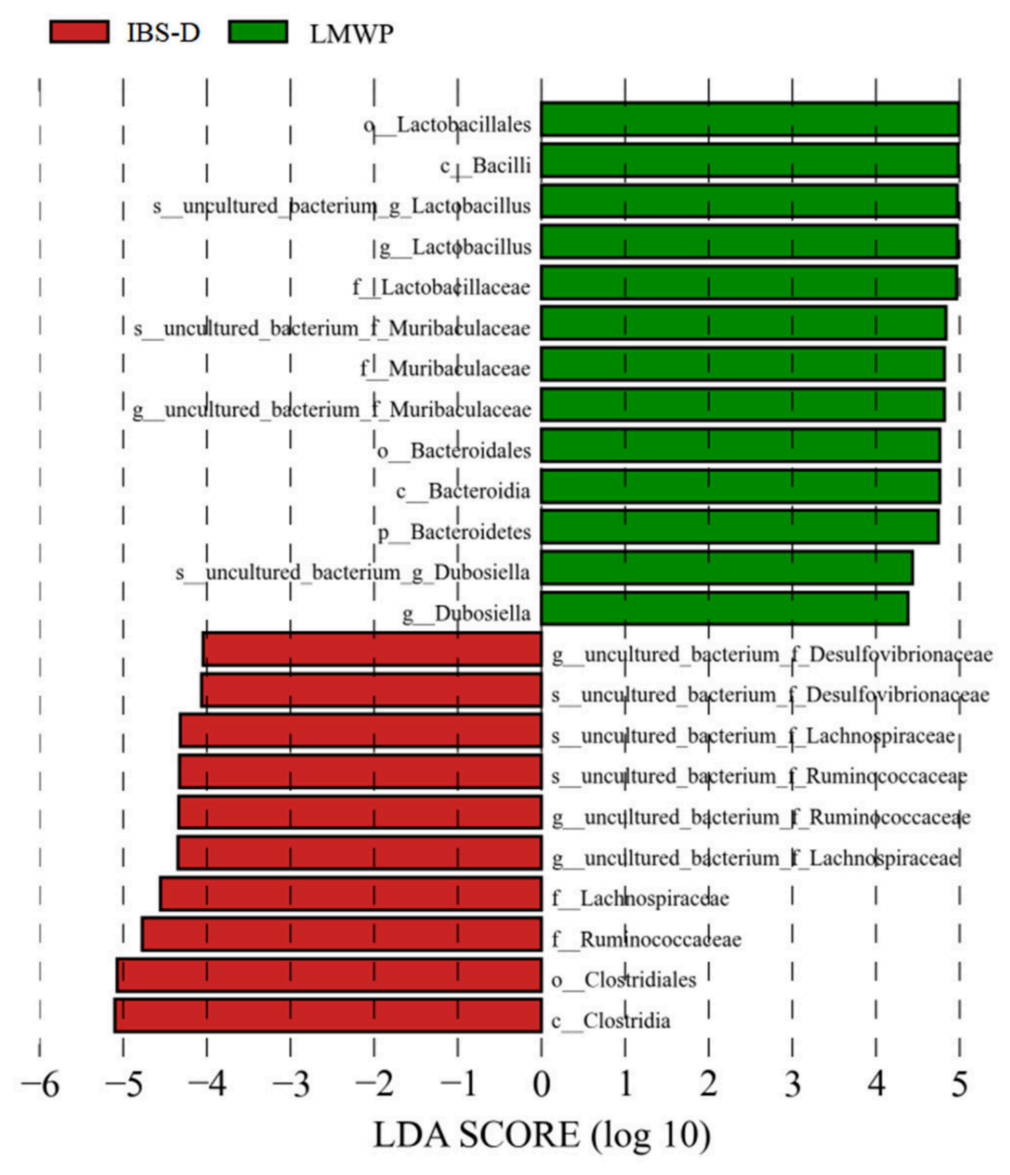
Genus-level analysis of significant intergroup differences in gut microbiota between LMWP and IBS-D groups.

LEfSe analysis at the genus level showed that LMWP treatment significantly increased the relative abundance of Lactobacillus and decreased that of uncultured_bacterium_f_Desulfovibrionaceae and uncultured_bacterium_f_Ruminococcaceae in the gut microbiota of IBS-D rats (Figure 9).

**Figure 10:**
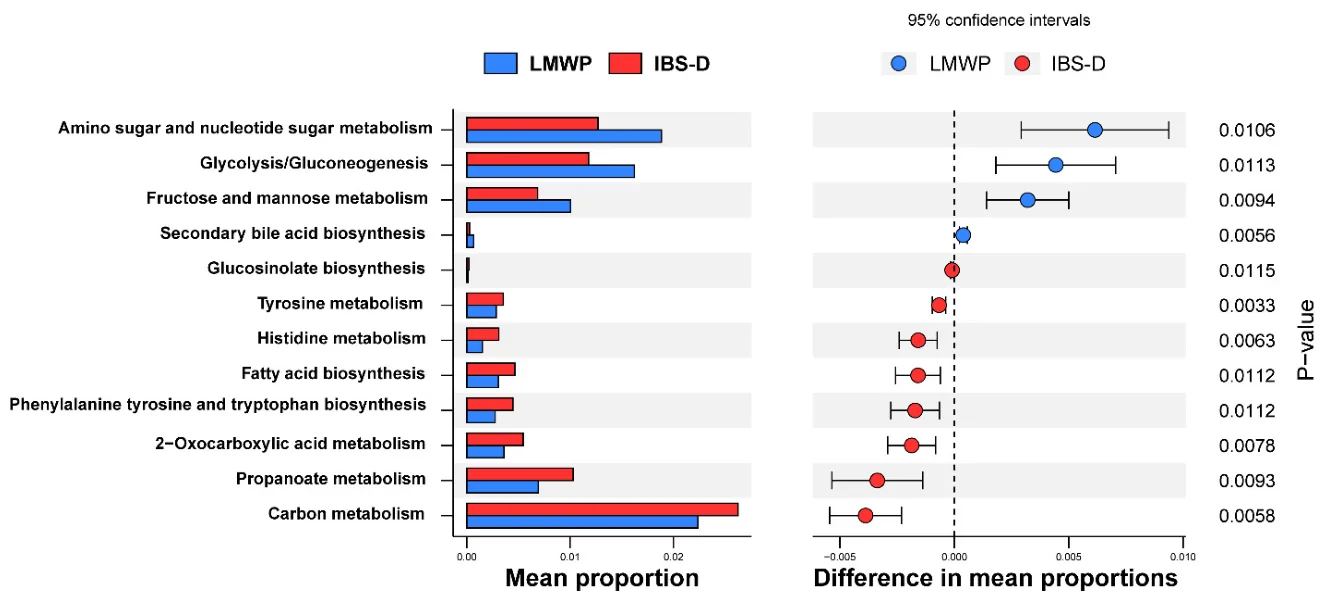
Functional prediction of altered intestinal microbiota in LMWP treatment group vs. IBS-D model group. Note: STAMP software (v2.1.3) was used for pairwise significance test of functional abundance among different samples, and pairwise T-test was performed among different groups with *p*-value threshold of 0.05 (*p* < 0.05 indicates significant difference).

Microbial metabolic functions were predicted via PICRUSt2 software based on 16S rRNA sequencing data, and LEfSe analysis was performed to compare the differential KEGG functional pathways at level 3 between the LMWP treatment group and IBS-D model group, with the aim of identifying the possible functional pathways involved in LMWP treatment for IBS-D. These pathways will be subject to experimental validation in follow-up research. Compared with the normal control group, IBS-D modeling significantly disturbed multiple intestinal flora metabolic pathways associated with intestinal barrier integrity, inflammatory response and nutrient metabolism, while the LMWP intervention markedly ameliorated these functional disorders with a regulatory effect comparable to trimebutine maleate. The differential functional pathways between the LMWP and IBS-D model groups were mainly concentrated in amino acid metabolism (histidine metabolism, phenylalanine, tyrosine and tryptophan biosynthesis, tyrosine metabolism), carbohydrate metabolism (2-oxocarboxylic acid metabolism, carbon metabolism, glycolysis/gluconeogenesis, etc.) and fatty acid metabolism (propanoate metabolism, fatty acid biosynthesis) (Figure 10). These findings demonstrate that the LMWP intervention not only regulated intestinal flora structure and composition, but also improved the functional homeostasis of gut microecology in IBS-D rats, with such functional alterations closely linked to the reshaped microbial community composition.

### 2.8. Effects of LMWPs on Serum Metabolomics in IBS-D Rats

**Figure 11:**
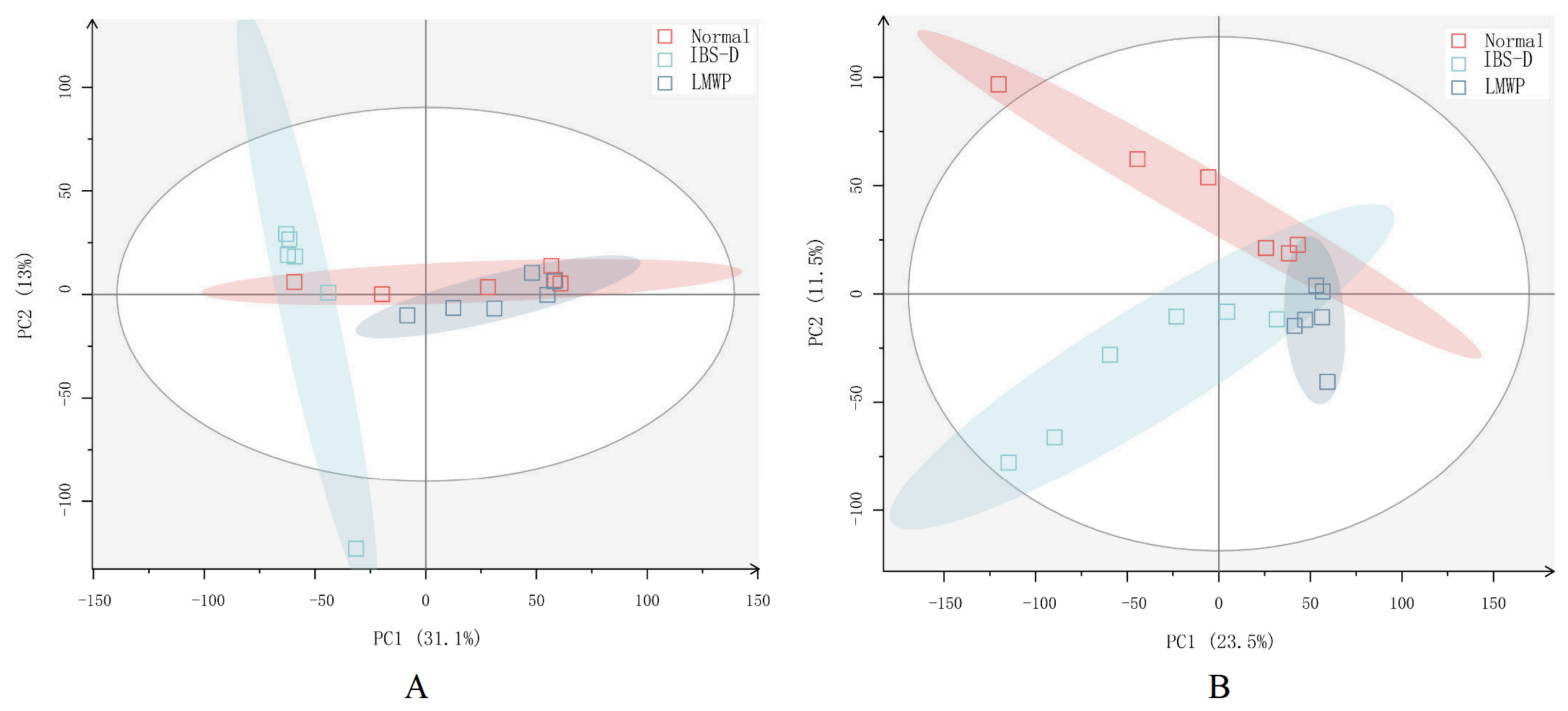
PCA for functional prediction of serum metabolites in LMWP-treated IBS-D rats: (**A**) positive ion mode and (**B**) negative ion mode.

Principal component analysis (PCA) was performed on serum metabolites identified in positive and negative ion modes to investigate the effects of LMWPs on serum metabolites in IBS-D rats, and PCA score plots (Figure 11A, positive ion mode; Figure 11B, negative ion mode) revealed distinct separation among the normal, IBS-D, and LMWP treatment groups under both ionization modes. Differential metabolites were screened using criteria of variable importance in projection (VIP) > 1 (OPLS-DA) and *p* < 0.05 (independent sample *t*-test), with VIP values reflecting the contribution of each metabolite to inter-group variation. In total, 80 IBS-D-related endogenous metabolites were identified in the IBS-D model group, and 27 of these metabolites were reversed after LMWP treatment (Table 2), verifying the regulatory effect of LMWP on serum metabolic profiles in IBS-D rats.

**Figure 12:**
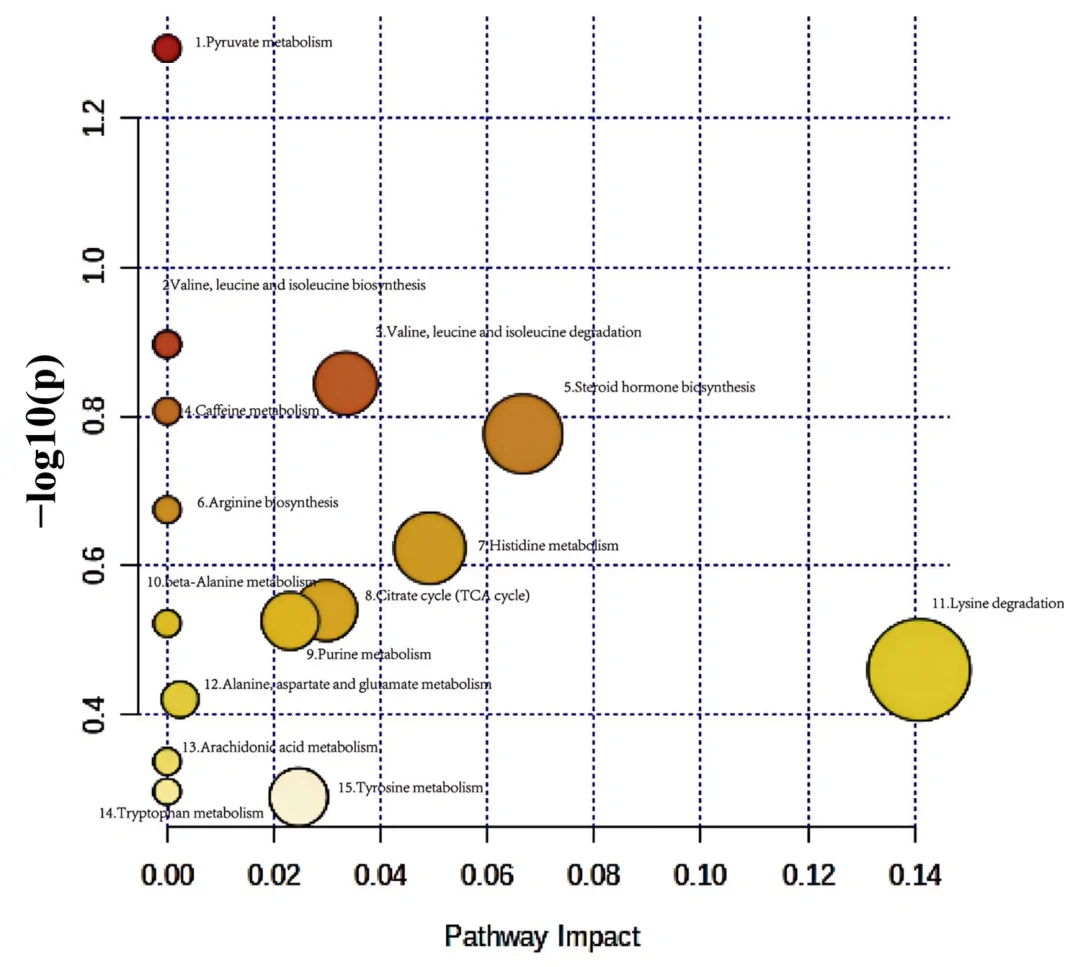
Metabolic pathway analysis. The vertical axis represents the negative logarithm (base 10) of the *p*-value for pathway enrichment analysis, and the horizontal axis denotes the impact value from pathway topology analysis; a larger −log10(*p*) indicates a more significant pathway alteration, while a higher impact value represents a greater topological weight of the corresponding pathway. Key disturbed pathways include pyruvate metabolism, ascorbate and aldarate metabolism, valine, leucine and isoleucine biosynthesis/degradation, caffeine metabolism, steroid hormone biosynthesis, arginine biosynthesis, histidine metabolism, the citrate cycle (TCA cycle), purine metabolism, beta-alanine metabolism, lysine degradation, alanine, aspartate and glutamate metabolism, arachidonic acid metabolism, tryptophan metabolism, and tyrosine metabolism.

To further excavate the potential information of differential metabolites, pathway analysis of the 27 differential metabolites was conducted using the online MetaboAnalyst platform by importing their KEGG IDs, and the pathway enrichment results are presented in Figure 12.

### 2.9. Effects of LMWPs on Fecal SCFA Contents in IBS-D Rats

**Figure 13:**
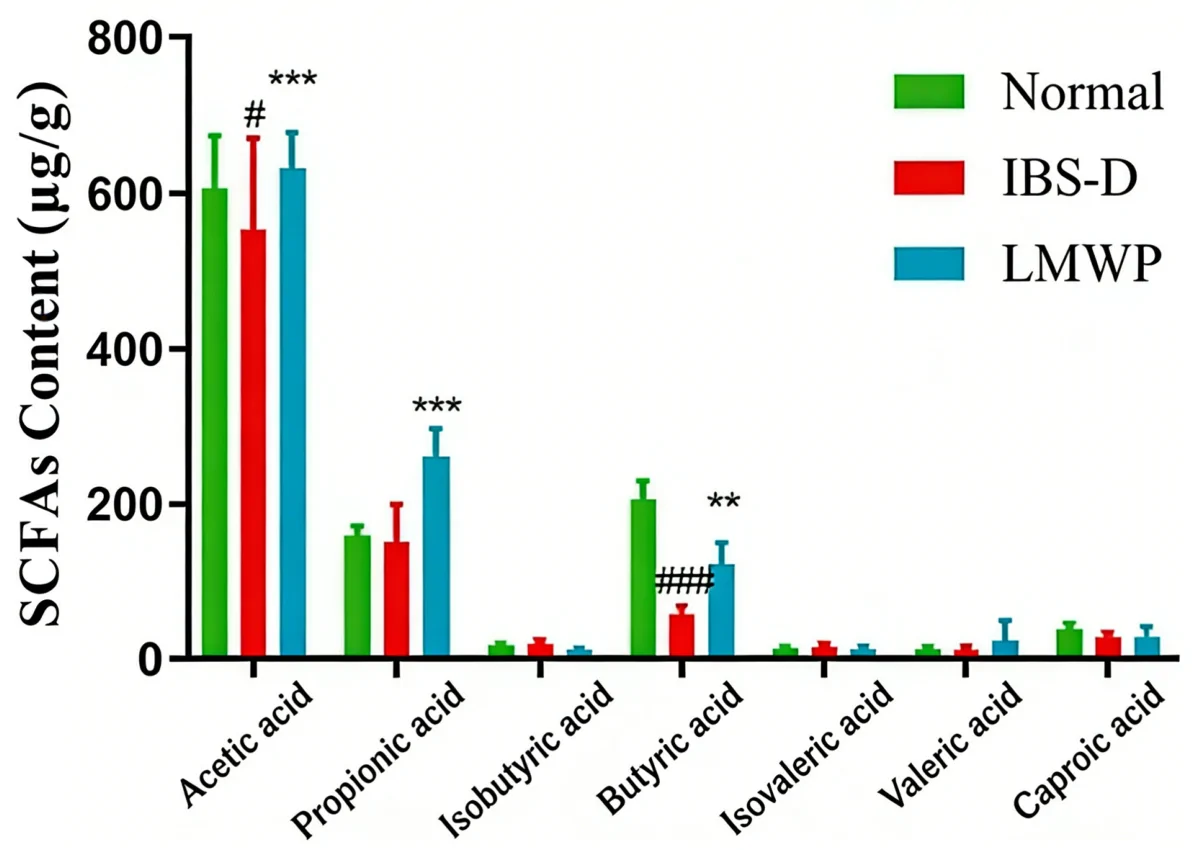
The effects of LMWP on fecal SCFA contents in IBS-D rats. ^#^ *p* < 0.05, ^###^ *p* < 0.001 vs. Normal; ** *p* < 0.01, *** *p* < 0.001 vs. IBS-D model. *n* = 6. Statistical significance was assessed using one-way ANOVA followed by Tukey’s post hoc test. ANOVA revealed significant effects for acetic acid, propionic acid, and butyric acid (all *p* < 0.0001), while no significant intergroup differences were detected for branched-chain and longer-chain SCFAs (all *p* > 0.05).

Furthermore, the effects of LMWPs on fecal short-chain fatty acid (SCFA) contents in IBS-D rats were determined (Figure 13). Compared with the normal group, the contents of acetic acid and butyric acid were significantly decreased in the IBS-D model group (*p* < 0.05). Compared with the IBS-D model group, the levels of acetic acid, butyric acid and propionic acid were markedly elevated in the LMWP treatment group (*p* < 0.001, *p* < 0.01, *p* < 0.001, respectively).

### 2.10. Multi-Omics Correlation Analysis Results

**Figure 14:**
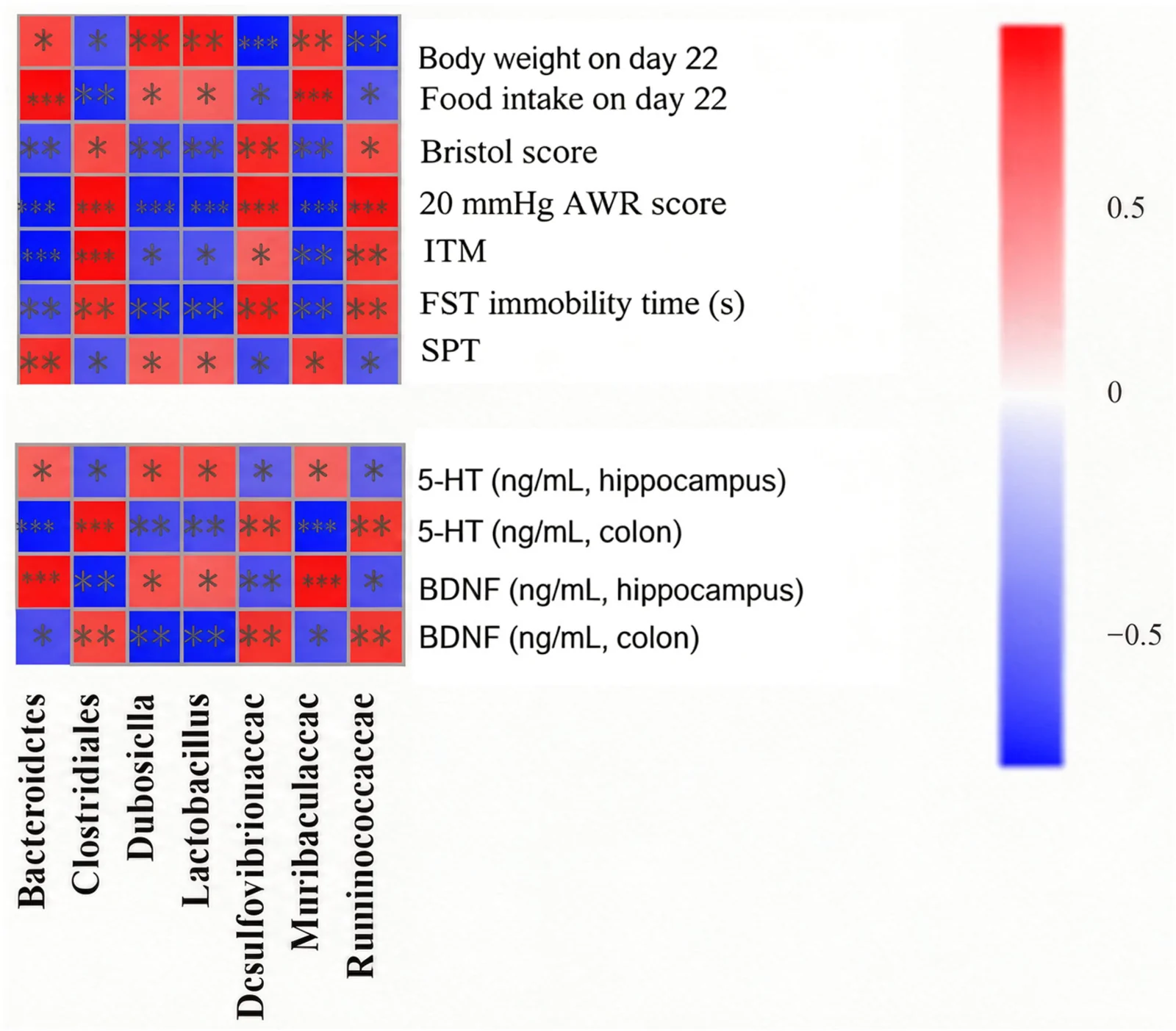
Heatmap analysis of correlations among IBS-D phenotype, molecular biological indicators, and dominant intestinal flora in rats. Color intensity represents the degree of correlation (red for positive correlation, blue for negative correlation); |r| ≥ 0.5, * *p* < 0.05, ** *p* < 0.01, and *** *p* < 0.001.

Comprehensive correlation analysis was performed to explore the associations among IBS-D phenotype, molecular biological indicators, differential metabolites, and intestinal flora. The correlation matrix was generated by calculating Spearman’s correlation coefficient. For the correlation between intestinal flora and phenotypic characteristics, Bristol stool score, 20 mmHg AWR score, ITM, and immobility time in FST were negatively correlated with Lactobacillus, uncultured_bacterium_f_Muribaculaceae, Dubosiella, and Bacteroidetes, but positively correlated with body weight on day 22, food intake, and sucrose preference. In contrast, uncultured_bacterium_f_Desulfovibrionaceae, uncultured_bacterium_f_Ruminococcaceae, and Clostridiales were negatively correlated with body weight on day 22, food intake, and sucrose preference, but positively correlated with Bristol stool score, 20 mmHg AWR score, ITM, and FST immobility time (Figure 14). Regarding the correlation between intestinal microbiota and molecular biological indicators, colonic concentrations of 5-HT and BDNF were negatively correlated with Lactobacillus, uncultured_bacterium_f_Muribaculaceae, Dubosiella, and Bacteroidetes, but positively correlated with hippocampal 5-HT and BDNF levels. Moreover, colonic 5-HT and BDNF concentrations were positively correlated with uncultured_bacterium_f_Desulfovibrionaceae, uncultured_bacterium_f_Ruminococcaceae, and Clostridiales, suggesting that the intestinal microbiota significantly affects host phenotypic and molecular biological indicators (Figure 14).

**Figure 15:**
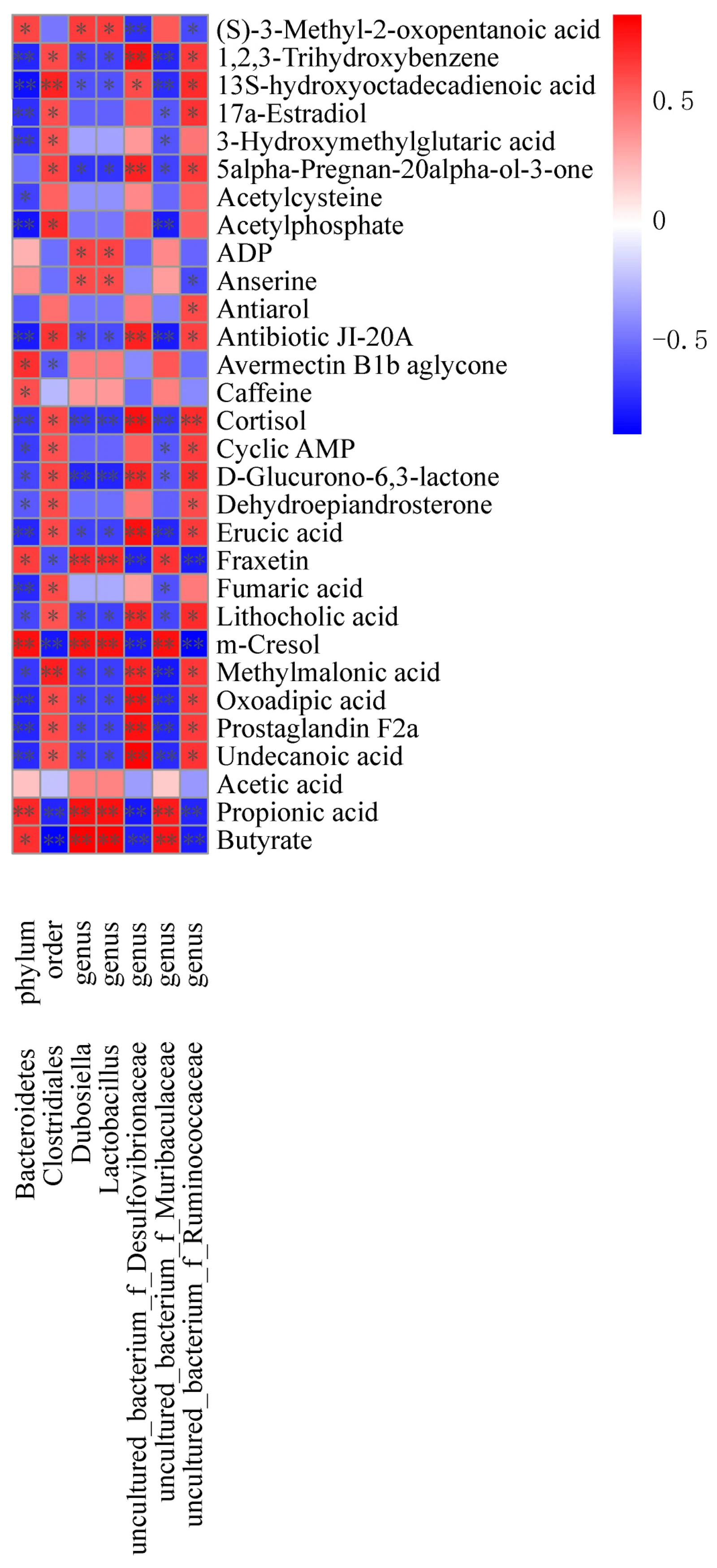
Heatmap of correlation analysis between serum metabolites, fecal SCFAs and dominant intestinal flora in rats. Color intensity represents the degree of correlation (red for positive correlation, blue for negative correlation); |r| ≥ 0.5, * *p* < 0.05, and ** *p* < 0.01.

**Figure 16:**
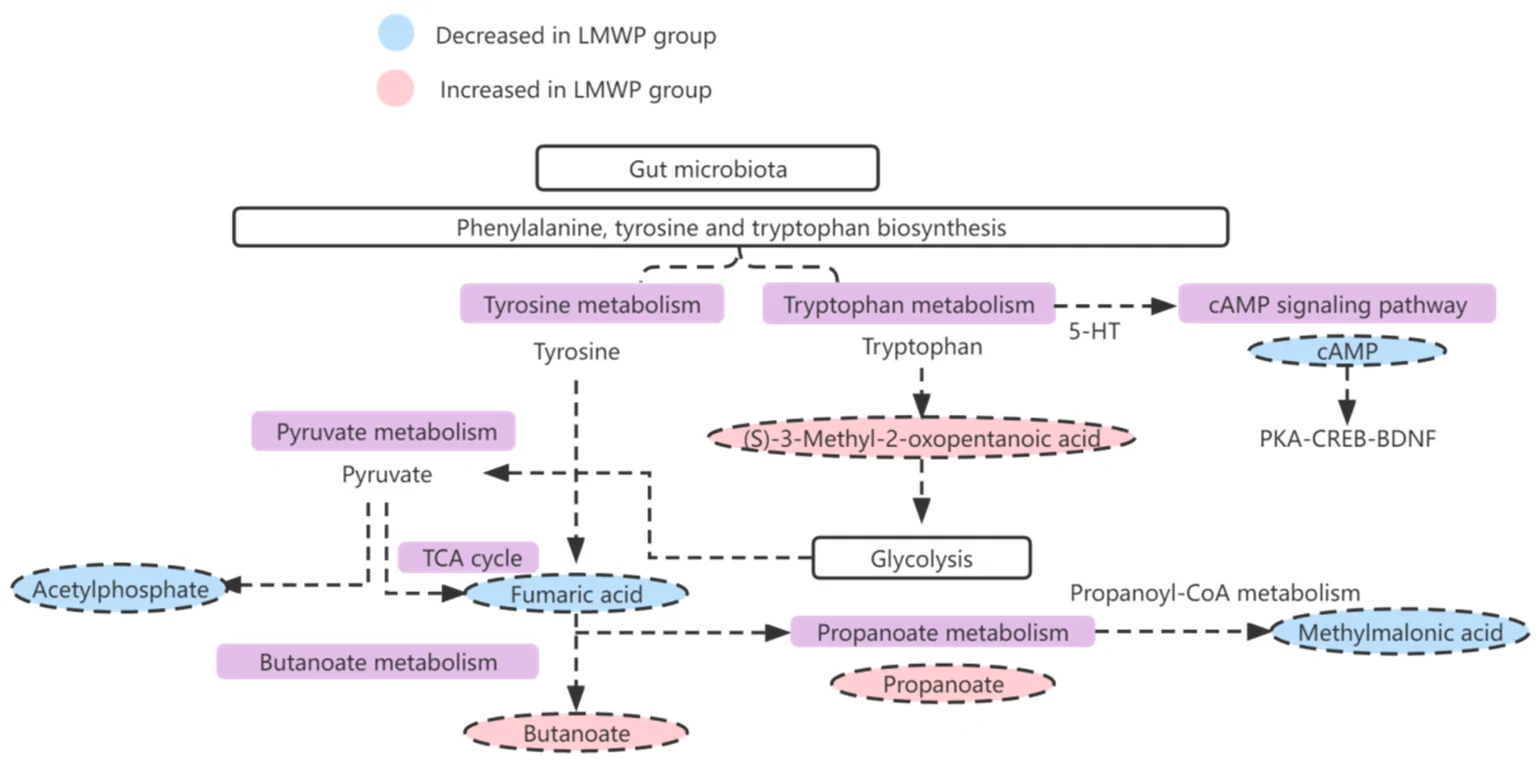
Network diagram of metabolic pathways related to LMWP intervention of IBS-D rats. Comparison between IBS-D and LMWP treatment groups; differential metabolites are upregulated (red) or downregulated (blue) in LMWP treatment group, and purple represents related metabolic pathways of metabolites in KEGG.

To further verify the association between LMWP-induced alterations in intestinal flora structure and the improvement in IBS-D-related metabolic disorders, Spearman correlation analysis was conducted between differential intestinal flora and metabolites (Figure 15). Combined with the metabolic pathway network diagram for LMWP-intervened IBS-D rats (Figure 16) and functional prediction analysis of intestinal flora between LMWP and IBS-D model groups (Figure 10), significant correlations between key metabolites and intestinal microorganisms were identified. Specifically, Lactobacillus was positively correlated with the tryptophan-related metabolite (S)-3-Methyl-2-oxopentanoic acid, propionic acid, and butyric acid, but negatively correlated with the propionic acid-related metabolite methylmalonic acid. Bacteroidetes was positively correlated with (S)-3-Methyl-2-oxopentanoic acid, propionic acid, and butyric acid, but negatively correlated with pyruvate and tyrosine metabolism-related metabolites (fumaric acid, acetylphosphate), the cAMP signaling pathway (cyclic AMP), and methylmalonic acid. Clostridiales was negatively correlated with propionic acid and butyric acid, but positively correlated with methylmalonic acid, fumaric acid, acetylphosphate, and cyclic AMP. Uncultured_bacterium_f_Ruminococcaceae was negatively correlated with (S)-3-Methyl-2-oxopentanoic acid, propionic acid, and butyric acid, but positively correlated with methylmalonic acid and cyclic AMP.

## 3. Discussion

Irritable bowel syndrome (IBS), particularly its diarrhea-predominant subtype (IBS-D), is a complex and debilitating functional gastrointestinal disorder (FGID) characterized by chronic abdominal pain, discomfort, and altered bowel habits [23]. A significant challenge in managing IBS-D lies in its high comorbidity with psychiatric disorders, including anxiety and depression, which can exacerbate symptoms and significantly impair patients’ quality of life [11,12]. This bidirectional pathological vicious cycle between intestinal dysfunction and emotional disturbances is largely mediated by the intricate gut–brain axis [24,25]. Traditional Chinese medicine has long recognized the therapeutic value of *Musca domestica* larvae, with historical records indicating their use for various ailments, including chronic malnutrition and indigestion [1]. Modern research has further elucidated the beneficial properties of peptides derived from *Musca domestica* larvae, such as anti-inflammatory, anti-diarrheal, and immunomodulatory effects [3,4]. Our previous study in mice confirmed LMWPs’ anti-diarrheal activity and Lactobacillus enrichment via PCR-DGGE [9]. The present study extends this by using an IBS-D with depression rat model, 16S rRNA sequencing, and integrated metabolomics to reveal systemic mechanisms. The higher dose (1.4 g/kg vs. 200 mg/kg) reflects interspecies scaling and model severity. Building upon our previous work demonstrating the anti-diarrheal activity of low-molecular-weight peptides (LMWPs) from *Musca domestica* larvae and their ability to regulate gut microbiota and the 5-HT signaling pathway [9,10], the present study aimed to comprehensively investigate the therapeutic efficacy and underlying molecular mechanisms of LMWPs in a rat model of IBS-D comorbid with depression, utilizing an integrated metabolomics and microbiomics approach to focus on its regulatory effects on intestinal microecology.

The successful establishment of the CACS-induced IBS-D rat model, exhibiting typical depressive behaviors, reduced locomotor activity, poor mental state, decreased appetite, body weight loss, severe diarrhea, and deteriorated fur appearance, is consistent with previously reported models that faithfully recapitulate both gastrointestinal dysfunction and concurrent psychological disturbances [26,27]. This model is crucial for exploring the pathogenesis and evaluating therapeutic interventions for IBS-D, a disorder closely linked to chronic stress and gut–brain axis dysregulation [28,29]. The marked reversal of these abnormal manifestations by the LMWP intervention, comparable to the positive control trimebutine maleate (TM), underscores its potent therapeutic potential. TM, a widely used drug for digestive system disorders, serves as a relevant benchmark for evaluating novel interventions in IBS-D [30]. The observed improvements in both physical and behavioral parameters suggest that LMWPs exert a comprehensive ameliorative effect on the multifaceted symptoms of IBS-D with depression.

A cornerstone of our findings is the significant impact of LMWPs on the gut microbiota. The IBS-D model group displayed a notable reduction in gut microbial alpha diversity (Shannon index) and a distinct separation in beta diversity (PCoA plot), indicative of a severely disrupted microbial community structure, a hallmark of IBS-D [21,22]. The LMWP intervention effectively reversed these dysbiotic patterns, restoring microbial diversity and shifting the overall community structure towards that of healthy controls. At the phylum level, the normalization of the Firmicutes/Bacteroidetes (F/B) ratio, which is often elevated in dysbiotic states and associated with various metabolic disorders [31], is a critical observation. Furthermore, LMWP treatment restored the balance of specific bacterial groups at the order and genus levels. The increase in beneficial bacteria such as Lactobacillus and the decrease in potentially pathogenic or pro-inflammatory groups like Clostridiales and certain Ruminococcaceae are particularly important. Lactobacillus species are well documented for their probiotic properties, including enhancing gut barrier function, modulating immune responses, and producing beneficial metabolites [32]. The ability of LMWPs to foster a more balanced and diverse gut microbiota composition is a key mechanism contributing to their therapeutic effects.

Beyond compositional changes, our study delved into the functional consequences of LMWPs’ microbiota modulation. PICRUSt software prediction and LEfSe analysis revealed that IBS-D modeling significantly perturbed multiple intestinal flora metabolic pathways, particularly those associated with amino acid, carbohydrate, and fatty acid metabolism. The LMWP intervention effectively ameliorated these functional disorders, suggesting the restoration of microbial metabolic homeostasis. The modulation of pathways involved in amino acid metabolism, such as histidine, phenylalanine, tyrosine, and tryptophan biosynthesis, is highly relevant to the gut–brain axis. Tryptophan, for instance, is a precursor for serotonin (5-HT), a crucial neurotransmitter involved in mood regulation and gut motility [33]. The observed improvements in depressive-like behaviors could be partly attributed to LMWPs’ ability to influence microbial tryptophan metabolism, thereby impacting central and enteric nervous system serotonin levels.

The serum metabolomics analysis provided a systemic perspective on LMWPs’ effects, revealing the normalization of numerous IBS-D-related endogenous metabolites and the modulation of several metabolic pathways. The identified changes in pathways such as pyruvate metabolism, valine/leucine/isoleucine biosynthesis/degradation, and steroid hormone biosynthesis indicate a broad impact on host metabolism. The multi-omics correlation analysis further strengthened these observations by establishing intricate links between specific gut microbes, host metabolites, and phenotypic outcomes. For example, the positive correlation between Lactobacillus and short-chain fatty acids (SCFAs) like propionic acid and butyric acid, as well as tryptophan-related metabolites, highlights a coherent mechanism. Short-chain fatty acids, which are produced by bacterial fermentation, can significantly enhance intestinal function, maintain gut microbial homeostasis, and regulate energy metabolism [34]. Moreover, they can influence brain function directly or indirectly through the gut–brain axis [35]. The significant increase in fecal acetic acid, propionic acid, and butyric acid levels in LMWP-treated rats provides direct evidence of enhanced SCFA production, which is likely a major contributor to the observed improvements in both gastrointestinal symptoms and depressive-like behaviors.

Integration of 16S rRNA sequencing, serum metabolomics, and fecal SCFA data revealed that LMWP reshapes the gut microbiota structure (enriching Bacteroidetes and Lactobacillus while suppressing Clostridiales), thereby coordinately regulating tryptophan metabolism and SCFA production. Correlation analyses showed that Lactobacillus was significantly positively correlated with tryptophan-related metabolites and butyrate, suggesting that LMWPs may enrich SCFA-producing beneficial bacteria. Systemically, this microbial remodeling may redirect tryptophan metabolism away from the pathological kynurenine pathway toward the 5-HT synthesis pathway, which potentially benefits central neurotransmitter status via humoral circulation. Concurrently, at the intestinal local level, SCFAs, particularly butyrate and propionate, can modulate TPH1 expression in enterochromaffin cells, correcting stress-induced excessive 5-HT secretion. Reigstad CS [36] pointed out that the gut microbiota acting through SCFAs is an important determinant of enteric 5-HT production and homeostasis. Therefore, the microbiota–metabolite shifts induced by LMWPs constitute a critical molecular bridge linking gut microbial remodeling to brain–gut axis functional restoration, providing a metabolic mechanistic explanation for the simultaneous alleviation of both intestinal symptoms and depressive/anxiety-like behaviors by LMWPs in IBS-D.

Our findings demonstrate that LMWPs exert opposing regulatory effects on the expression of 5-HT1AR, PKA, CREB, and BDNF in the hippocampus and colon, upregulating this pathway in the hippocampus while downregulating it in the colon. A comparable phenomenon has been reported in a rat model of IBS following resveratrol intervention [37]. The implications are that low hippocampal 5-HT levels represent a core pathological basis for depression and anxiety; accordingly, LMWPs activate the hippocampal 5-HT1A–PKA–CREB–BDNF cascade, conferring antidepressant and anxiolytic effects. In contrast, abnormally elevated colonic 5-HT drives hypermotility and visceral hypersensitivity, whereas LMWPs normalize colonic 5-HT levels, thereby attenuating this pathway and relieving diarrhea and abdominal pain. Critically, the abrogation of these effects by a 5-HT1A receptor antagonist further substantiates the pivotal role of this pathway in mediating the therapeutic actions of LMWPs.

Several limitations of this study should be acknowledged: (1) 16S sequencing was performed on only three rats per group, a relatively small sample size that may affect the statistical power for detecting differences in gut microbiota; (2) the PICRUSt functional predictions are inferred from reference databases rather than direct metatranscriptomic evidence, and thus require validation by metagenomic or metatranscriptomic approaches; (3) the correlation analyses reveal associations only and do not establish causal relationships.

## 4. Materials and Methods

### 4.1. Chemicals and Reagents

*Musca domestica* larvae were obtained from Xufang Chinese Herb Management Co., Ltd. (Anguo, China). The rabbit anti-5-HT1AR antibody was purchased from Affinity BioReagents (Golden, CO, USA); the rabbit anti-PKA antibody was sourced from Abcam (Cambridge, UK); the rabbit anti-BDNF antibody was acquired from Proteintech Group (Wuhan, China). Trimebutine maleate was purchased from Yuanye Biotechnology Co., Ltd. (Shanghai, China). The rat 5-HT ELISA kit and rat BDNF ELISA kit were obtained from EnzymeLink Biotechnology Co., Ltd. (Shanghai, China). The total RNA extraction kit, RNA reverse transcription kit, and RT-qPCR kit were purchased from Akerui Bioengineering Co., Ltd. (Changsha, China).

### 4.2. Preparation of Low-Molecular-Weight Peptides from Musca domestica Larvae

Low-molecular-weight peptides (LMWPs) derived from *Musca domestica* larvae were prepared via ultrafiltration centrifugation, following the protocol described in our team’s prior patent [9]. The raw *Musca domestica* larvae material was taxonomically authenticated by a qualified botanist upon acquisition. Freshly frozen larvae were thawed, weighed, and homogenized in 3 volumes (*w*/*v*) of distilled water, followed by filtration through a 200-mesh sieve to remove debris. The filtrate was subjected to ultrasonic extraction at 14 kHz and 800 W for 40 s (5 cycles) to enhance peptide release. After centrifugation at 12,000× *g* for 25 min at 4 °C, the supernatant was heat-treated at 90 °C for 12 min to precipitate macromolecular proteins (e.g., albumin). Following ice-water quenching for 10 min, re-centrifugation at 12,000× *g* for 25 min at 4 °C was performed to clarify the solution. The resulting supernatant was fractionated using a 30 kDa ultrafiltration membrane to collect peptides with molecular weights < 30 kDa, which were then lyophilized to obtain a dry powder. The molecular weight distribution of the resultant peptides was verified using SDS-PAGE with a rapid gel preparation kit (Servicebio, Wuhan, China).

### 4.3. Animals and Experimental Design

**Figure 17:**
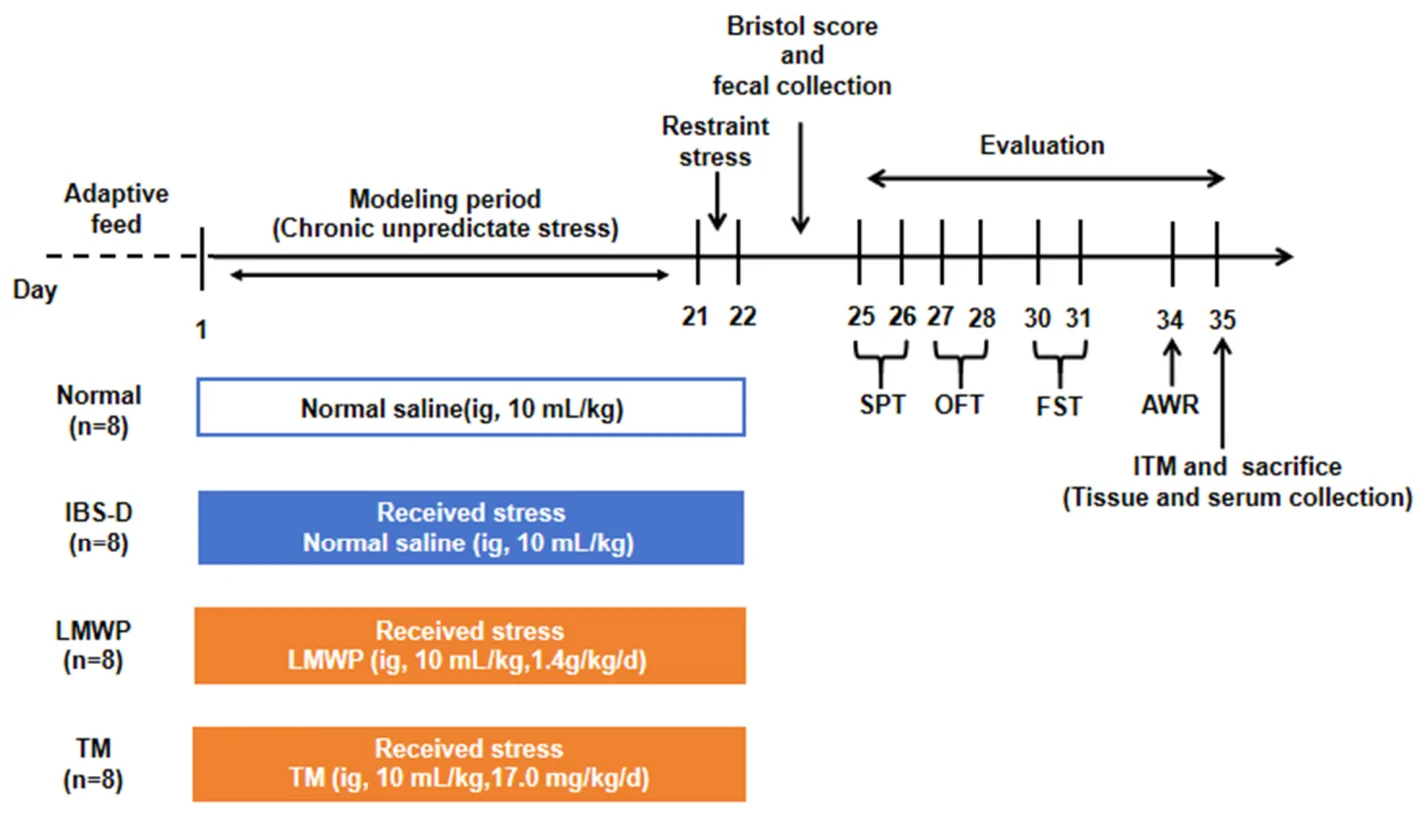
A timeline of experimental the procedures. Normal saline was given by gavage to the normal control group and the IBS-D with depression model group. The positive control group received trimebutine maleate (17.0 mg/kg/d), and the experimental group received *Musca domestica* larvae LMWPs (1.4 g/kg); both drugs were dissolved in normal saline and administered intragastrically. SPT, sucrose preference test; OFT, open-field test; FST, forced swim test; AWR, abdominal withdrawal reflex; ITM, intestinal tract motility test.

Thirty-two male SD rats (180 ± 10 g) were acclimatized for one week with free access to food and water under controlled conditions (room temperature 22 ± 2 °C, normal light/dark cycle). After acclimatization, rats were randomly divided into four groups (*n* = 8 each): IBS-D with depression model group (negative control), trimebutine maleate group (positive control, 17 mg/kg), *Musca domestica* larvae LMWP treatment group (experimental, 1.4 g/kg), and normal control group. All animals received the respective treatments via oral gavage. With the exception of the normal control group, the remaining three groups underwent model induction according to the protocol previously established by our research group for the rat model of irritable bowel syndrome (IBS-D) [27]. The experimental timeline is as follows (Figure 17).

### 4.4. Assessment of IBS-D with Depression Phenotypes

This section comprehensively evaluates IBS-D with depression phenotypes in rats through five key dimensions: body weight and food intake, fecal traits, visceral sensitivity, colonic motility and depression-like behaviors.

#### 4.4.1. Body Weight and Food Intake

Body weight was measured at a fixed time daily using a calibrated electronic balance; for food intake assessment, a specified amount (X grams) of standard rodent chow was provided to each cage at 10:00 a.m., spilled chow was collected and weighed separately, the remaining chow (Y grams) was weighed at 10:00 a.m. the next day, and daily food intake was calculated by the following formula: Daily food intake = X (provided amount) − Y (remaining amount) − spilled amount (unit: g).

#### 4.4.2. Fecal Traits

Rats were individually housed in metabolic cages lined with filter paper for a 4 h observation period, during which the number of fecal pellets was recorded and fecal consistency was evaluated using a modified Bristol Stool Scale adapted for rodents—Grade 1: separate hard lumps (nut-like, dry); Grade 2: lumpy sausage-shaped; Grade 3: sausage-shaped with surface cracks; Grade 4: smooth, soft sausage or snake-like; Grade 5: soft blobs with distinct edges; Grade 6: mushy stool with ragged edges; Grade 7: watery diarrhea with no solid structure.

#### 4.4.3. Visceral Sensitivity

After a 24 h fast (with free access to water), a latex balloon catheter was inserted 3 cm into the rectum via the anus, then graded balloon distensions (10, 20, 30, and 40 mmHg) were applied—each lasting 30 s with 180 s intervals to avoid tissue adaptation—and behavioral responses were blindly scored by two independent observers using the abdominal withdrawal reflex (AWR) scale: Score 1: no observable behavioral response; Score 2: mild abdominal muscle contraction; Score 3: visible elevation of the abdominal wall; Score 4: severe body arching with pelvic elevation.

#### 4.4.4. Colonic Motility

After a 30 min acclimatization period, rats were placed in individual Perspex restraint chambers for 1 h, and the total number of fecal pellets expelled during this period was counted as an indicator of colonic transit function.

#### 4.4.5. Depression-like Behaviors

For the forced swim test (FST), rats were placed in transparent glass cylinders (40 cm in height × 18 cm in diameter) filled with 23 cm deep water maintained at 25 ± 1 °C, and after a 2 min adaptation period, immobility time (defined as floating without active swimming or climbing movements) was recorded over the subsequent 4 min using a video tracking system. The open-field test (OFT) was performed in a 100 cm × 100 cm × 40 cm open arena. Rats were transferred to the test chamber one day in advance for environmental acclimatization. During the test, each rat was placed into the arena with its back facing the operator, and its movement trajectories within 5 min were recorded by a video tracking system. All animals were tested sequentially. Behavioral parameters including the total travel distance, central zone travel distance and residence time in the central zone of rats in each group were analyzed using analytical software. For the sucrose preference test (SPT), rats were subjected to 24 h food and water deprivation, then presented with two bottles containing 1% sucrose solution and tap water, and after a 1 h habituation period, fluid consumption was measured over 24 h, with sucrose preference rate calculated by the following formula: Sucrose preference rate = (Sucrose solution consumption/Total fluid consumption) × 100%.

### 4.5. Enzyme-Linked Immunoassay (ELISA)

After serial thawing at −20 °C and 4 °C, hippocampal and colonic tissues were homogenized in saline and centrifuged at 13,000 rpm, 4 °C for 10 min. The resulting supernatant was assayed for BDNF and 5-HT concentrations using rat ELISA kits (Enzyme Link, Shanghai, China) and operated according to the instructions.

### 4.6. Real-Time Quantitative PCR Detection

Total RNA was extracted from hippocampal and colonic tissues using Trizol reagent (Accurate Biology Co., Ltd., Changsha, China), with quantification and purity assessment performed on a NanoDrop 2000 spectrophotometer (Thermo Fisher Scientific, Waltham, MA, USA), where all samples demonstrated optimal A260/A280 ratios ranging from 1.8 to 2.0. Subsequently, 1 μg of total RNA was reverse-transcribed into cDNA using a PrimeScript RT reagent kit (Accurate Biology Co., Ltd., Changsha, China) incorporating a gDNA eraser step to eliminate genomic DNA contamination. Quantitative PCR was carried out in 20 μL reactions comprising 10 μL SYBR Green Master Mix (Accurate Biology Co., Ltd., Changsha, China), 0.4 μL of each gene-specific primer (10 μM), 2 μL cDNA template, and 7.2 μL nuclease-free water. Amplification was conducted on a StepOnePlus Real-Time PCR System (Applied Biosystems, Foster City, CA, USA) under the following conditions: initial denaturation at 95 °C for 30 s, followed by 40 cycles of 95 °C for 10 s and 60 °C for 30 s, with a final melting curve analysis to verify amplification specificity. Gene expression analysis of 5-HT1AR, BDNF, PKA, and CREB was normalized to the reference gene GAPDH, and relative quantification was determined using the 2^−ΔΔCT^ method with custom-designed primers (see Table 3 for details).

### 4.7. Detection of Intestinal Microecological Diversity

On the 22nd day of the experiment, colonic feces of rats in the normal control group, negative control group, positive control group and experimental (LMWP) group were collected under sterile conditions, with three rats in each group. Then, the microbial genomic DNA in the fecal samples of each group was extracted using the PowerSoil DNA Isolation Kit in strict accordance with the kit instructions. The V3-V4 region of 16S rRNA was amplified by PCR with primers 338F (5′-ACTCCTACGGGAGGCAGCA-3′) and 806R (5′-GGACTACHVGGGTWTCTAAT-3′) under the following conditions: initial denaturation at 98 °C for 1 min, followed by denaturation at 98 °C for 10 s, annealing at 50 °C for 30 s, extension at 72 °C for 60 s, and final extension at 72 °C for 5 min. After amplification, the samples were purified using the MinElute Gel Extraction Kit (Qiagen, Düsseldorf, Germany), and samples with a length of 400–450 bps were selected to generate sequencing libraries using the NEB Next Ultra DNA Library Prep Kit from Illumina (NEB, Ipswich, MA, USA) according to the manufacturer’s protocol, followed by high-throughput pyrosequencing on the Illumina MiSeq/HiSeq2500 platform (Beijing Biomarker Technology Co., Ltd., Beijing, China). Subsequently, QIIME 1.8.0 software was used to identify Operational Taxon Units (OTUs), the RDP classifier with Greengenes database (version 13.5) was used for taxonomic annotation of each representative sequence, and QIIME software was also used to perform weighted principal coordinate analysis (PCoA) based on UniFrac distance. Alpha diversity, which reflects the species diversity within a single sample, was measured by the Shannon index, while beta diversity was analyzed by PCoA to show the differences in species diversity between samples. Taxonomic analysis was conducted by comparing OTU representative sequences with microbial reference databases to count the species composition of intestinal flora at each level (phylum, class, order, family, genus, species), generate abundance tables using QIIME software, and plot the species structure using R language tools. LEfSe analysis was performed with Metastats software (v2.0) to identify biomarker species with significant differences between groups, and PICRUSt2 software was used to predict the function of intestinal flora based on 16S rRNA and reference sequence databases.

### 4.8. Serum Non-Targeted Metabolomics Analysis

Serum samples were harvested from rats in the normal control, negative control, and *Musca domestica* larvae small-molecule peptide-treated experimental groups under sterile conditions. For serum sample pretreatment, samples were thawed at 4 °C; a 100 µL aliquot of each sample was mixed with 400 µL −20 °C methanol, vortexed for 60 s, and centrifuged at 12,000 rpm (4 °C) for 10 min. Supernatants were vacuum-concentrated to dryness, reconstituted with 150 µL 80% methanol containing 4 ppm 2-chlorophenylalanine, and filtered through a 0.22 µm membrane, and quality control (QC) samples were prepared by pooling 20 µL of each test sample for LC-MS analysis. Chromatographic separation was performed on a Thermo Vanquish UPLC system (Thermo Fisher Scientific, Waltham, MA, USA) equipped with an ACQUITY UPLC^®^ HSS T3 column (1.8 µm, 2.1 × 150 mm), with an autosampler temperature of 8 °C, a column temperature of 40 °C, a flow rate of 0.25 mL/min, an injection volume of 2 μL, mobile phases consisting of 0.1% formic acid–water (A2)/0.1% formic acid–acetonitrile (B2) for positive ion mode and 5 mM ammonium formate–water (A3)/acetonitrile (B3) for negative ion mode, and gradient elution programmed as 0–1 min (2% B2/B3), 1–9 min (2–50% B2/B3), 9–12 min (50–98% B2/B3), 12–13.5 min (98% B2/B3), 13.5–14 min (98–2% B2/B3), and 14–20 min (2% B2 for positive mode; 14–17 min, 2% B3 for negative mode). Mass spectrometric analysis was conducted on a Thermo Q Exactive mass spectrometer (Thermo Fisher Scientific, Waltham, MA, USA) with an ESI source operating in positive and negative ion modes, with spray voltage of 3.50 kV (positive) and 2.50 kV (negative), sheath gas of 30 arb, auxiliary gas of 10 arb, a capillary temperature of 325 °C, full-scan resolution of 70,000 (*m*/*z* 81–1000), HCD fragmentation at 30 eV, and dynamic exclusion for MS/MS analysis. Raw data were converted to mzXML format via Proteowizard (v3.0.8789), processed using XCMS (R v3.3.2) for peak picking, filtering and alignment with parameters bw = 2, ppm = 15, peakwidth = c (5,30), mzwid = 0.015, mzdiff = 0.01 and centWave method, generating a data matrix of *m*/*z*, retention time and peak intensity (6058 positive, 11,245 negative precursor molecules) that was exported to Excel and normalized by peak area for cross-sample comparison. Processed data were imported into SIMCA for unsupervised PCA (dimensionality reduction) and supervised OPLS-DA (intergroup differentiation), with model validity assessed by R2X (>0.5 indicates good fit) and Q2 via 200 permutation tests to exclude overfitting. Metabolites with OPLS-DA variable importance in projection (VIP) > 1 and *p* < 0.05 (independent sample *t*-test) were defined as significant differential metabolites, which were identified by accurate mass (error < 30 ppm) and MS/MS fragment matching against HMDB, Metlin, MassBank and LipidMaps databases, followed by metabolic pathway enrichment analysis using MetaboAnalyst 5.0 online software.

### 4.9. Fecal SCFA Content Analysis

For targeted determination of intestinal short-chain fatty acids (SCFAs) in fecal samples, standard stock solutions of seven individual SCFAs were prepared by accurately weighing reference substances, dissolving them in anhydrous ethanol, and diluting to volume, followed by preparation of serial mixed working standard solutions with gradient concentrations as required. For sample extraction, 100 mg of frozen fecal samples was mixed with 1000 μL ethanol solution containing 0.5% (*v*/*v*) concentrated HCl, vortexed homogenously, extracted ultrasonically for 30 min, and centrifuged at 14,000 r/min for 10 min; the supernatant was filtered through an organic membrane prior to GC-MS analysis. GC conditions were set as follows: TG-WAXMS capillary column (30 m × 0.25 mm × 0.25 μm), helium as carrier gas at a flow rate of 1.0 mL/min, an injection port temperature of 250 °C with splitless mode. The temperature program was as follows: initial temperature held at 50 °C for 2 min, ramped to 120 °C at 15 °C/min, 170 °C at 5 °C/min, and 240 °C at 15 °C/min with a 3 min hold. MS conditions employed an electron impact (EI) ion source with 70 eV electron energy, operating in full-scan and SIM modes. Chromatographic peak areas and retention times were extracted using Dionex Chromeleon 7.2 software to plot standard curves, and SCFA contents in samples were calculated using the following formula: SCFAs content in sample (μg/g) = [extract concentration (μg/mL) × extract volume (mL)]/sample weight (g).

### 4.10. Multi-Omics Correlation Analysis

To assess potential relationships among behavioral, molecular, and metabolic variables, Spearman’s rank correlation analysis was conducted between key phenotypic indicators (including abdominal withdrawal reflex scores and sucrose preference), neurobiological markers (BDNF and 5-HT levels), and differentially expressed metabolites. Correlations with an absolute coefficient value |r| > 0.5 and a *p*-value less than 0.05 were deemed statistically significant. The resulting association patterns were graphically represented as a heatmap generated using the pheatmap package in R (version 4.2.0).

The criteria for selecting taxa included in the correlation analysis are as follows: (1) taxa were selected from those showing statistically significant differences in relative abundance between groups (identified by the Kruskal–Wallis test with Dunn’s post hoc test, FDR-adjusted q < 0.05); (2) taxa were further filtered to include only those with a mean relative abundance > 0.1% across all samples to exclude rare taxa with unreliable estimates; (3) for genus-level analysis, the top differentially abundant genera (based on LDA scores from LEfSe analysis, LDA > 2.0) were selected for correlation analysis.

### 4.11. Statistical Analysis

Statistical analyses were performed using SPSS 26.0 (IBM, Armonk, NY, USA), with all data expressed as the mean ± standard deviation (SD). The normality of data distribution was evaluated using the Shapiro–Wilk test, and homogeneity of variance was assessed via Levene’s test. For comparisons among multiple groups, one-way analysis of variance (ANOVA) was employed, followed by Tukey’s honestly significant difference (HSD) post hoc test for detailed group-wise comparisons. Data involving repeated measures, such as longitudinal behavioral observations, were analyzed using two-way ANOVA. A probability value of *p* < 0.05 was considered statistically significant. PERMANOVA was performed based on weighted UniFrac distance matrices using the vegan package in R (adonis2 function) with 999 permutations to ensure full reproducibility of the statistical method. Graphical representations of the data were generated using GraphPad Prism version 8.0 (GraphPad Software, San Diego, CA, USA). For alpha diversity indices and taxonomic relative abundances, data were analyzed using the Kruskal–Wallis H-test, followed by Dunn’s post hoc test for pairwise comparisons. Multiple testing correction was performed using the Benjamini–Hochberg false discovery rate (FDR) method, and statistical significance was set at adjusted q < 0.05.

## 5. Conclusions

In conclusion, our integrated multi-omics study provides compelling evidence that LMWPs derived from *Musca domestica* larvae exert significant therapeutic effects on IBS-D comorbid with depression in rats. This is achieved through comprehensive modulation of the gut microecology, encompassing the restoration of gut microbial diversity and composition, the enhancement in beneficial microbial metabolic functions, the normalization of host serum metabolic profiles, and the increase in fecal short-chain fatty acid levels. These multifaceted actions collectively contribute to the alleviation of both gastrointestinal symptoms and depressive-like behaviors, primarily through the intricate interplay within the gut–brain axis. This study highlights LMWPs as a promising natural product for the management of IBS-D with depression. Future investigations should focus on isolating and characterizing the specific active peptide components within LMWPs, elucidating their precise molecular targets, and conducting rigorous clinical trials to validate these promising preclinical findings in human patients.

## Figures and Tables

**Table 1:** Open-field performance parameters of rats in each group.

Group	Total Distance (cm)	Distance in Center (cm)	Time in Center (s)
Normal	2984.69 ± 1248.05	231.18 ± 105.50	8.46 ± 3.67
Model	2804.48 ± 736.06	35.96 ± 11.30 ^###^	1.03 ± 0.55 ^###^
TM	2882.14 ± 1211.80	142.875 ± 35.30 *	2.97 ± 0.65
LMWP	3035.24 ± 1379.38	144.05 ± 21.98 *	6.60 ± 1.90 **

Note: ^###^ *p* < 0.001 vs. Normal; * *p* < 0.05 vs. Model; ** *p* < 0.01 vs. Model.

**Table 2:** Analysis of significant differential metabolites in rat serum.

Metabolites	Normal vs. IBS-D			IBS-D vs. LMWP		
	*p*.Value	VIP	Change	*p*.Value	VIP	Change
Cyclic AMP	0.0136	1.261	↑	0.0126	1.013	↓
Erucic acid	0.0216	3.373	↑	0.000737	3.711	↓
Fumaric acid	0.00897	3.321	↑	0.0163	2.932	↓
1,2,3-Trihydroxybenzene	0.0282	3.55	↑	0.000701	4.02	↓
ADP	0.000278	2.733	↓	0.0325	1.675	↑
m-Cresol	0.00865	1.11	↓	0.000118	1.23	↑
Prostaglandin F2a	0.0184	3.305	↑	0.000554	3.613	↓
Acetylcysteine	0.000831	3.552	↑	0.00263	2.942	↓
Undecanoic acid	0.015	3.538	↑	0.000652	3.778	↓
Lithocholic acid	0.0049	1.938	↑	0.00733	1.426	↓
17a-Estradiol	0.00333	1.739	↑	0.0227	1.014	↓
Cortisol	0.0109	3.505	↑	0.0007	3.637	↓
Antibiotic JI-20A	0.0238	3.318	↑	0.000556	3.719	↓
Acetylphosphate	0.0169	1.405	↑	0.0116	1.235	↓
Avermectin B1b aglycone	0.00649	1.556	↓	0.0323	1.05	↑
Antiarol	0.00186	3.473	↑	0.00381	2.913	↓
5alpha-Pregnan-20alpha-ol-3-one	0.0451	1.216	↑	0.0472	1.084	↓
Fraxetin	0.0272	1.343	↓	0.0143	1.191	↑
(S)-3-Methyl-2-oxopentanoic acid	0.00292	1.782	↓	0.00677	1.074	↑
3-Hydroxymethylglutaric acid	0.028	2.824	↑	0.00672	2.941	↓
Caffeine	0.0211	1.511	↓	0.0365	1.052	↑
Oxoadipic acid	0.0226	3.62	↑	0.000562	4.035	↓
Dehydroepiandrosterone	0.000744	2.024	↑	0.0234	1.247	↓
D-Glucurono-6,3-lactone	0.0267	1.32	↑	0.016	1.231	↓
13S-hydroxyoctadecadienoic acid	0.00595	1.49	↑	0.000579	1.462	↓
Methylmalonic acid	0.0351	1.226	↑	0.0408	1.024	↓
Anserine	0.0306	1.315	↓	0.0369	1.115	↑

**Table 3:** Primer sequences used for quantitative real-time PCR.

Gene Symbol	Full Name	Accession Number	Primer Sequence (5′ → 3′)	Product Size (bp)
5-HT1AR	5-Hydroxytryptamine Receptor 1A	NM_012585.2	F: CCCTGGCATCTACTTCATCATC	121
			R: AGTCCATGCCATTGACTTCTTC	
BDNF	Brain-Derived Neurotrophic Factor	NM_001270630.1	F: AGCTGAGCGAGTGCAAA	189
			R: GATCGCCAGCCAATTCTCT	
PKA	Protein Kinase A Catalytic Subunit	NM_001270861.1	F: TGGAGACGGAGAAGGTGATG	156
			R: GGTCGGTACTTGGCATAGGA	
CREB	cAMP Response Element-Binding Protein	NM_001277928.1	F: CAGTGGCATCCACGATCAG	142
			R: GGCTCTGGGATCTTGACTCC	
GAPDH	Glyceraldehyde-3-Phosphate Dehydrogenase	NM_017008.4	F: GACATGCCGCCTGGAGAA	135
			R: AGCCCAGGATGCCCTTTAGT	

## Data Availability

The original contributions presented in this study are included in the article. Further inquiries can be directed to the corresponding author.
